# Characterization of type I and type II diacylglycerol acyltransferases from the emerging model alga *Chlorella zofingiensis* reveals their functional complementarity and engineering potential

**DOI:** 10.1186/s13068-019-1366-2

**Published:** 2019-02-11

**Authors:** Xuemei Mao, Tao Wu, Yaping Kou, Ying Shi, Yu Zhang, Jin Liu

**Affiliations:** 1 Laboratory for Algae Biotechnology & Innovation, College of Engineering, Peking University, Beijing, 100871 China; 2 BIC-ESAT, College of Engineering, Peking University, Beijing, 100871 China

**Keywords:** *Chlorella zofingiensis*, Diacylglycerol acyltransferase, Trait improvement, Functional characterization, Transcription factor, Triacylglycerol

## Abstract

**Background:**

The green alga *Chlorella zofingiensis* has been recognized as an industrially relevant strain because of its robust growth under multiple trophic conditions and the potential for simultaneous production of triacylglycerol (TAG) and the high-value keto-carotenoid astaxanthin. Nevertheless, the mechanism of TAG synthesis remains poorly understood in *C. zofingiensis*. Diacylglycerol acyltransferase (DGAT) is thought to catalyze the committed step of TAG assembly in the Kennedy pathway. *C. zofingiensis* genome is predicted to possess eleven putative DGAT-encoding genes, the greatest number ever found in green algae, pointing to the complexity of TAG assembly in the alga.

**Results:**

The transcription start site of *C. zofingiensis DGAT*s was determined by 5′-rapid amplification of cDNA ends (RACE), and their coding sequences were cloned and verified by sequencing, which identified ten *DGAT* genes (two type I *DGAT*s designated as *CzDGAT1A* and *CzDGAT1B*, and eight type II *DGAT*s designated as *CzDGTT1* through *CzDGTT8*) and revealed that the previous gene models of seven *DGAT*s were incorrect. Function complementation in the TAG-deficient yeast strain confirmed the functionality of most DGATs, with CzDGAT1A and CzDGTT5 having the highest activity. In vitro DGAT assay revealed that CzDGAT1A and CzDGTT5 preferred eukaryotic and prokaryotic diacylglycerols (DAGs), respectively, and had overlapping yet distinctive substrate specificity for acyl-CoAs. Subcellular co-localization experiment in tobacco leaves indicated that both CzDGAT1A and CzDGTT5 were localized at endoplasmic reticulum (ER). Upon nitrogen deprivation, TAG was drastically induced in *C. zofingiensis*, accompanied by a considerable up-regulation of *CzDGAT1A* and *CzDGTT5*. These two genes were probably regulated by the transcription factors (TFs) bZIP3 and MYB1, as suggested by the yeast one-hybrid assay and expression correlation. Moreover, heterologous expression of *CzDGAT1A* and *CzDGTT5* promoted TAG accumulation and TAG yield in different hosts including yeast and oleaginous alga.

**Conclusions:**

Our study represents a pioneering work on the characterization of both type I and type II *C. zofingiensis DGAT*s by systematically integrating functional complementation, in vitro enzymatic assay, subcellular localization, yeast one-hybrid assay and overexpression in yeast and oleaginous alga. These results (1) update the gene models of *C. zofingiensis DGAT*s, (2) shed light on the mechanism of oleaginousness in which CzDGAT1A and CzDGTT5, have functional complementarity and probably work in collaboration at ER contributing to the abundance and complexity of TAG, and (3) provide engineering targets for future trait improvement via rational manipulation of this alga as well as other industrially relevant ones.

**Electronic supplementary material:**

The online version of this article (10.1186/s13068-019-1366-2) contains supplementary material, which is available to authorized users.

## Background

*Chlorella zofingiensis*, a freshwater green alga also referred to as *Chromochloris zofingiensis* or *Muriella zofingiensis*, has been considered as a potential astaxanthin producer alternative to *Haematococcus pluvialis* because of its capacity of synthesizing astaxanthin and growing robustly under multiple trophic conditions for high biomass concentration [1–7]. It is also capable of accumulating high level of triacylglycerol (TAG), the most energy-dense lipid, and emerges as a promising feedstock for biodiesel [4, 5, 8–10]. Moreover, oleic acid (C18:1), which is thought to be beneficial to biodiesel quality [11], is rich in lipids of *C. zofingiensis* [5]. The integrated production of TAG with astaxanthin, a value-added keto-carotenoid with strong anti-oxidative activity and broad applications, has the potential to be achieved in *C. zofingiensis* [5, 10], and is believed to be a promising approach toward offsetting algal biodiesel production cost. Another approach to bring down production cost is genetic engineering for trait improvement, e.g., TAG modulation, which relies on a better understanding of the molecular mechanism for TAG biosynthesis [12, 13].

TAG biosynthesis is believed to be mainly from two pathways, acyl-CoA-dependent Kennedy pathway and acyl-CoA-independent pathway, which has been well documented in higher plants [14, 15]. The former pathway involves a series of acylation steps mediated by various acyltransferases. Among them, diacylglycerol acyltransferase (DGAT) catalyzes the last, also committed, step by transferring the acyl moiety from an acyl-CoA to the *sn*-3 position of diacylglycerol (DAG), and plays a critical role in contributing TAG synthesis [15]. To date, three types of DGATs have been well characterized in higher plants, namely, the membrane-bound type I (DGAT1) and type II (DGAT2 or DGTT) and the soluble type III (DGAT3) [16]. Intriguingly, higher plants generally contains a single DGAT2 [17], while algae have multiple ones, e.g., five for *Chlamydomonas reinhardtii* [18] and *Chlorella pyrenoidosa* [19], four for *Phaeodactylum tricornutum* [20], and eleven for *Nannochloropsis oceanica* [21, 22]. This large difference may point to more complex regulation of TAG synthesis in algae. *C. reinhardtii* represents the most studied alga for DGAT characterization [23–26]. Notably, the study of Liu et al. [25], conducting for the first time both in vitro and in vivo assays of multiple type II DGATs, supported that they have distinctive substrate preferences and work in concert spatially and temporally to synthesize diverse TAG species in *C. reinhardtii*. Nevertheless, *C. reinhardtii* is a model alga and not considered as an oleaginous organism for lipid production, driving the research interest to industrially relevant algae such as *N. oceanica* [27–29] and *P. tricornutum* [30]. Recently, the genome of *C. zofingiensis* was sequenced and annotated [31], providing the genomic foundation for the characterization of *DGAT*s. It has been observed that *C. zofingiensis DGAT*s responded differentially upon various abiotic stress conditions [31, 32]. However, many remain unknown as to whether they are functional, their subcellular localization, substrate preferences and engineering potential in lipid production. Furthermore, it has been proposed that certain DGATs may catalyze astaxanthin esterification in *H. pluvialis* [33], further driving us to dissert the function and biology of *C. zofingiensis* DGATs.

The genome of *C. zofingiensis* is predicted to contain eleven putative DGAT-encoding genes [31], but their gene models have not been verified. In the present study, we cloned their full-length coding sequences with confirmed transcription start sites, and updated the incomplete gene models presented in Roth et al. [31]. A total of ten *DGAT*s were verified and renamed: two type I *DGATs* designated as *CzDGAT1A* and *CzDGAT1B*, and eight type II *DGAT*s designated as *CzDGTT1* through *CzDGTT8*. A pioneering characterization of both type I and type II *C. zofingiensis DGAT*s was conducted by integrating the analyses of in silico, subcellular localization, in vitro, and heterologous expression in various hosts. CzDGAT1A and CzDGTT5, which showed the highest acyltransferase activity, are ER-localized and have distinctive substrate preferences. We discuss their roles in TAG metabolism and biotechnological implications.

## Results

### Cloning and bioinformatics analysis of *C. zofingiensis DGAT*s

*Chlorella zofingiensis* genome is predicted to contain eleven putative *DGAT* genes, two of which, Cz03g14080 and Cz09g23020, are exactly the same [31]. Nevertheless, their gene models have not been verified. Through further checking the chromosomes Chr03 and Chr09 where Cz03g14080 and Cz09g23020 locate, respectively, we found that the two chromosomes share a long fragment sequence (35-kb in common), which covers the two above-mentioned genes (Additional file 1: Figure S1). Considering that the chromosomes were assembled from the fragments of whole genome rather than of the isolated chromosome [31], a wrong assembly of this part may occur for Chr03 or Chr09. To validate this, primer pairs were designed for Chr03 (f1 + r1) and Chr09 (f2 + r1): f1 and f2 are specific to Chr03 and Chr09, respectively, while r1 locates at the site with common sequence (Additional file 1: Figure S1). PCR results using genomic DNA as the template showed that the former primer pair (f1 + r1) gave the expected band, while the latter one (f2 + r1) had no band (Additional file 1: Figure S1), indicating the incorrect assembly of Chr09. In this context, Cz03g14080 and Cz09g23020 should be at the same locus and assigned with the same name. o obtain the full-length coding sequence of the ten genes, here a 5′ rapid amplification of cDNA ends (RACE) experiment was performed to determine the start codon and 5′ untranslated region (5′ UTR) sequence prior to cloning. Then, the full-length coding sequences were cloned and sequenced, which were renamed and deposited in NCBI Genbank with accession numbers: two type I *DGAT*s designated as *CzDGAT1A* and *CzDGAT1B*, and eight type II *DGAT*s designated as *CzDGTT1* through *CzDGTT8* (Additional file 2: Table S1). This is so far the highest dose of *DGAT*s reported in green algae. All except *CzDGTT6* contain introns ranging from 3 to 9 (Additional file 1: Figure S2). This is in consistence with *N. oceanica* in which two type II *DGAT*s are intron-less [22]. Comparison between gene models of *DGAT*s presented in Roth et al. [31] and our confirmed ones revealed that the previous gene models of seven *DGAT*s were incorrect (Additional file 1: Figure S2). In this context, the genome annotation for *C. zofingiensis* from Roth et al. [31] remains yet to be improved.

To gain insights into the evolutionary relationship between *C. zofingiensis* DGATs and other orthologs, a cladogram was reconstructed using MEGA6 [34] based on the multiple sequences from higher plants, animals, yeast, and algae (Additional file 1: Figure S3). CzDGAT1A and CzDGAT1B, clustered with the algal type I DGAT orthologs, are distinct from type II DGATs. Of the eight type II *C. zofingiensis* DGATs, CzDGTT1 and CzDGTT2 are highly close to *C. reinhardtii* DGTT1, CzDGTT4 is close to *C. reinhardtii* DGTT4, CzDGTT5 is close to *C. reinhardtii* DGTT3, CzDGTT6 through CzDGTT8 are somewhat close to *C. reinhardtii* DGTT2, while CzDGTT3 is kindly distant from other type II DGATs. By contrast, *N. oceanica* DGATs are somewhat distant from *C. zofingiensis* DGATs. Although containing no intron, CzDGTT6 is grouped well within the CzDGTTs in the phylogenetic tree (Additional file 1: Figure S3), indicating that it may be of vertical origin rather than from horizontal gene transfer. Several DGATs from *C. reinhardtii* and *N. oceanica* have been well characterized both in vitro and in vivo [25, 27–29], providing insights into the function of their corresponding homologs in *C. zofingiensis*. When analyzed with TMHMM Server 2.0, *C. zofingiensis* DGATs are predicted to contain ~ 9 transmembrane domains (Additional file 1: Figure S4). The analysis of *C. zofingiensis* DGATs using PredAlgo, a new multi-subcellular localization prediction tool dedicated to algae [34], together with TargetP and ChloroP, suggests their localization other than in chloroplast or mitochondrion (Additional file 2: Table S2). This is in consistence with the subcellular localization prediction of DGATs from the green alga *C. reinhardtii* [25].

### TAG synthesis and transcriptional expression of *C. zofingiensis DGAT*s in *C. zofingiensis*

**Fig. 1:**
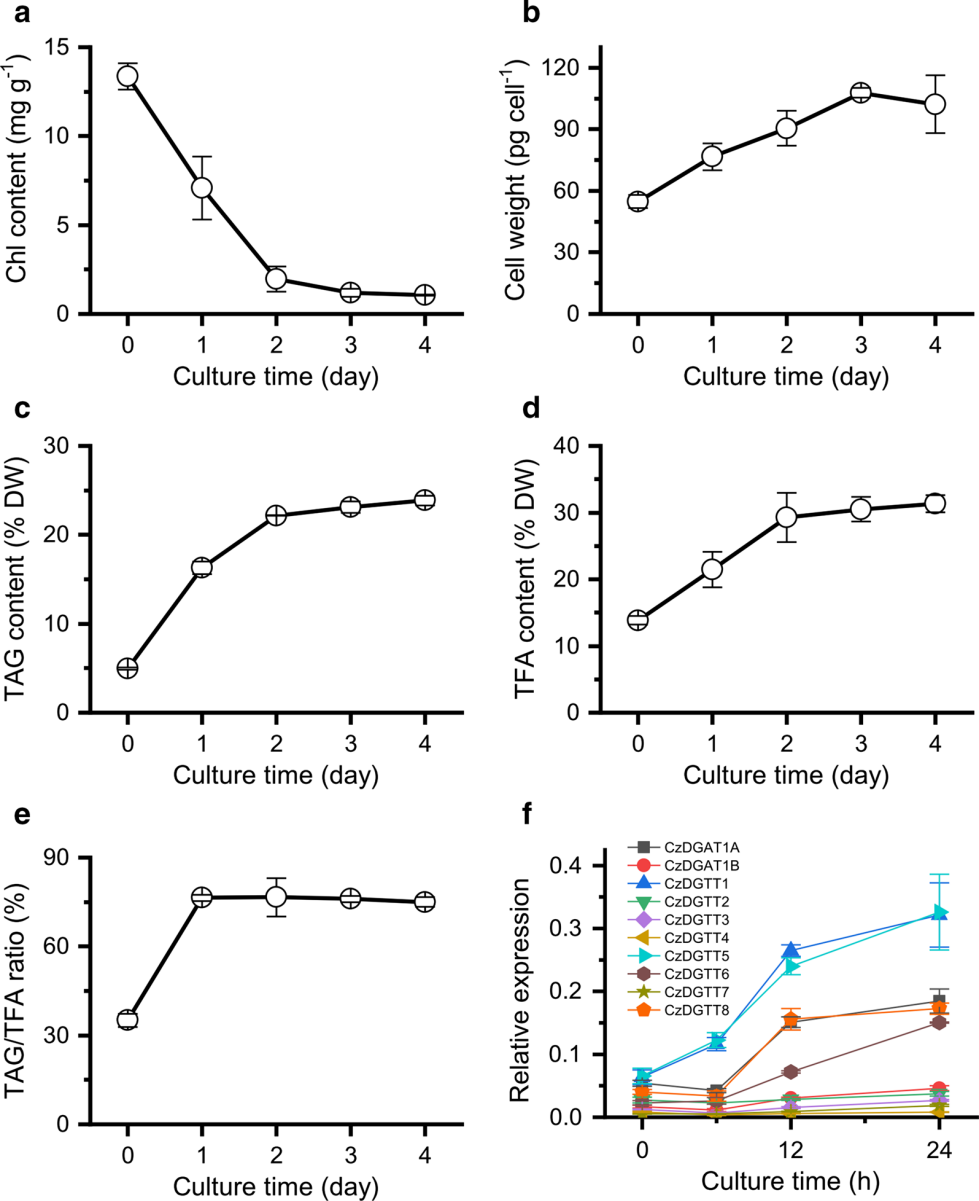
Growth, lipid changes and *DGAT* expression in *C. zofingiensis* in response to ND. **a** Chlorophyll content. **b** Cell weight. **c** TAG content. **d** TFA content. **e** TAG/TFA ratio. **f** Transcriptional level of *DGAT* genes. The data are expressed as mean ± SD (*n* = 3)

TAG synthesis and accumulation in algae can be triggered by abiotic stresses such as the deprivation of nutrients particularly nitrogen [35]. Obviously, upon nitrogen deprivation (ND), the chlorophyll content of *C. zofingiensis* showed a sharp decrease (Fig. 1a), implying severe growth impairment. By contrast, the per cell weight increases gradually (Fig. 1b), accompanied by a considerable increase in TAG, total fatty acids (TFA) and TAG/TFA ratio (Fig. 1c–e). The fatty acid composition of TAG was also impacted considerably by ND: C18:1 increased while the polyunsaturated fatty acids including C18:3n3, C16:3 and C16:4 decreased (Table 1).

**Table 1:** Fatty acid profile of TAG in *C. zofingiensis* in response to ND

Fatty acid	Culture time (day)				
	0	1	2	3	4
C16:0	18.7 ± 0.0	18.0 ± 0.4	16.7 ± 0.4	16.0 ± 0.1	15.7 ± 0.1
C16:1	1.9 ± 0.0	1.2 ± 0.0	1.2 ± 0.0	1.3 ± 0.0	1.4 ± 0.0
C16:2	2.8 ± 0.2	2.8 ± 0.1	3.4 ± 0.0	3.6 ± 0.0	3.7 ± 0.1
C16:3	8.5 ± 0.3	6.2 ± 0.1	5.3 ± 0.0	5.2 ± 0.0	5.2 ± 0.1
C16:4	4.0 ± 0.0	1.5 ± 0.1	0.9 ± 0.0	0.7 ± 0.0	0.5 ± 0.2
C18:0	2.4 ± 0.3	3.4 ± 0.1	3.5 ± 0.0	3.5 ± 0.2	3.4 ± 0.1
C18:1	19.3 ± 0.2	31.8 ± 0.7	34.8 ± 0.1	35.5 ± 0.1	36.2 ± 0.3
C18:2	16.2 ± 0.2	19.3 ± 0.0	20.8 ± 0.2	21.1 ± 0.3	21.0 ± 0.4
C18:3 n3	22.7 ± 0.1	13.6 ± 0.2	11.6 ± 0.1	11.3 ± 0.0	11.1 ± 0.2
C18:3 n6	1.4 ± 0.0	1.1 ± 0.0	1.0 ± 0.0	1.0 ± 0.00	1.0 ± 0.0
C18:4	2.1 ± 0.0	1.2 ± 0.0	0.9 ± 0.0	0.8 ± 0.0	0.8 ± 0.0

The fatty acid profile (%) is expressed as mean ± SD (*n *= 3)

To correlate TAG synthesis with the expression of *DGAT*s, the time-resolved transcript levels of the ten *DGAT*s were determined by real-time quantitative PCR (Fig. 1f). *CzDGAT1A*, *CzDGTT1*, *CzDGTT5* and *CzDGTT8* had a higher basal transcript levels (0 h of ND) and were all considerably up-regulated by ND, yet their expression patterns differed: the up-regulation of *CzDGTT1* and *CzDGTT5* began earlier and was greater than that of *CzDGAT1A* and *CzDGTT8*. *CzDGTT6* also showed an up-regulation in response to ND. By contrast, the other five *DGAT* genes remained relatively stable at the transcript level. These results suggest the involvement of *CzDGAT1A*, *CzDGTT1*, *CzDGTT5*, *CzDGTT6* and *CzDGTT8* in ND-induced TAG synthesis.

### Functional complementation of *C. zofingiensis DGAT*s in the TAG-deficient yeast strain *S. cerevisiae* H1246

**Fig. 2:**
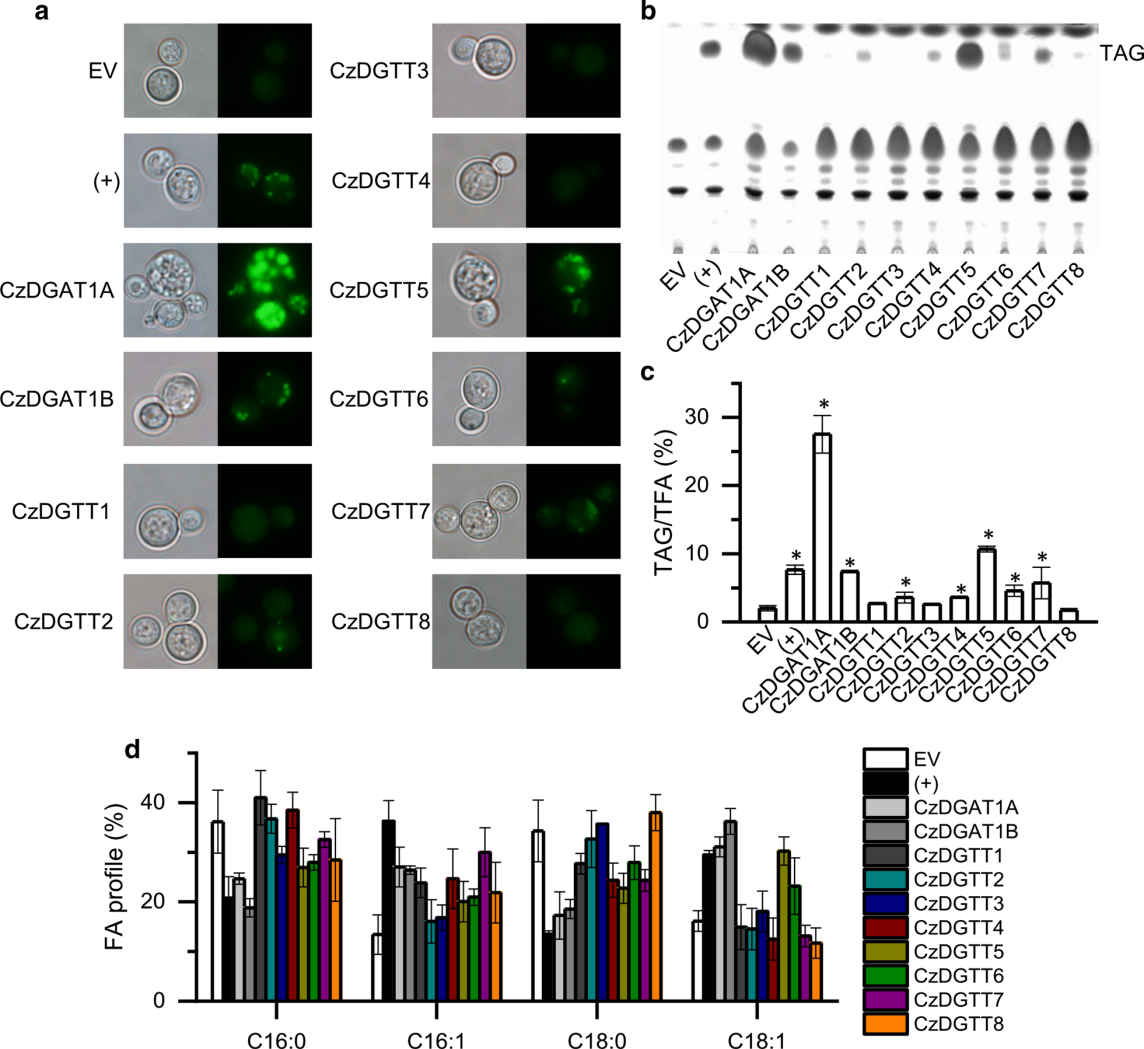
Functional complementation of *DGAT*s in the TAG-deficient yeast strain *S. cerevisiae* H1246. **a** BODIPY staining of H1246 cells expressing various *C. zofingiensis DGAT*s. Left, bright field; right, fluorescent field. Green fluorescence indicates the BODIPY-bound lipid droplets. **b** TLC analysis of TAG. **c** Quantification of TAG, expressed as TAG/TFA ratio. **d** Fatty acid profile of TAG. EV (empty vector)-expressing H1246 strain was used as the negative control and *C. reinhardtii* DGTT1-expressing H1246 strain was used as the positive control (+). The data in (**c**) and (**d**) are expressed as mean ± SD (*n* = 3). Asterisks indicate the significant difference compared to EV (*t*-test, *P* < 0.01)

To validate the acyltransferase function of the ten putative *C. zofingiensis* DGATs, they were each introduced into the TAG-deficient *S. cerevisiae* strain H1246 [36], a system widely used for functional complementation of DGATs of heterologous origins including higher plants and algae [20, 25, 28, 29, 37–39]. The H1246 expressing the empty vector (EV) and *CrDGTT1*, a type II *DGAT* from *C. reinhardtii* with confirmed activity [25], were used as the negative and positive control, respectively. All transformants were subjected to staining with the fluorescence dye BODIPY (Fig. 2a), TLC-based neutral lipid analysis (Fig. 2b) and TAG quantification using GC–MS (Fig. 2c). Obviously, similar to EV control, the H1246 cells expressing *CzDGTT1*, *CzDGTT3* or *CzDGTT8* produced a trace amount of TAG, suggesting their null DGAT function in yeast. By contrast, other seven DGATs were functional as they, similar to the positive control (+), made H1246 to produce more TAG, yet to different extents. Notably, CzDGAT1A and CzDGTT5 were most functional, as evidenced by the highest TAG levels which accounted for 27.5% and 10.7% of TFA, respectively (Fig. 2c). The expression of functional *C. zofingiensis DGAT*s also impacted the fatty acid profile of TAG in H1246: enhanced unsaturated fatty acids of C16:1 and C18:1 at the expense of saturated fatty acids of C16:0 and C18:0 (Fig. 2d).

It is worth noting that the yeast H1246 contains only four fatty acids (C16:0, C16:1, C18:0 and C18:1), much less than *C. zofingiensis* does [5]. The lack of certain fatty acids may affect the results of functional complementation for *C. zofingiensis DGAT*s in H1246, which can be evaluated by feeding exogenous free fatty acid (FFA). Here, we tested the effect of three FFAs including C18:2 and C18:3n3 that are present in *C. zofingiensis*, and C20:5 that is a high-value omega-3 fatty acid, on TAG content and fatty acid composition. We hypothesized that if a DGAT had activity on the exogenously fed FFA, the FFA should be incorporated into TAG in the *DGAT*-expressing H1246, and verse vice. In the EV control lacking acyltransferases, the fed FFAs were not detected in TAG in spite of their presence in polar lipids (PL) (Table 2). Similarly, the H1246 strains expressing *CzDGTT3*, *CzDGTT4*, and *CzDGTT6*–*CzDGTT8* each had no detected exogenous FFAs incorporated into TAG (Table 2), indicative of their null function on these fatty acids. By contrast, CzDGAT1A, CzDGAT1B, and CzDGTT5 all had activity on these fatty acids, as evidenced by their presence in significant percentage in TAG (Table 2). Intriguingly, CzDGTT1, which showed no activity in the functional complementation (Fig. 1c), incorporated the exogenous C18:2 and C18:3 into TAG and promoted TAG accumulation (Table 2), suggesting the activity of CzDGTT1 on the two fatty acids. On the other hand, CzDGTT2 showed no activity on C18:2 or C18:3 but was active on C20:5 (Table 2).

**Table 2:** TAG and PL analysis in DGAT-expressing H1246 transformants fed with exogenous FFAs

Strains	TAG (μg OD^−1^)	C18:2				C18:3				C20:5			
		TAG (μg OD^−1^)	C18:2 in TAG (%)	PL (μg OD^−1^)	C18:2 in PL (%)	TAG (μg OD^−1^)	C18:3 in TAG (%)	PL (μg OD^−1^)	C18:3 in PL (%)	TAG (μg OD^−1^)	C20:5 in TAG (%)	PL (μg OD^−1^)	C20:5 in PL (%)
EV	0.10 ± 0.01	0.21 ± 0.01	–	1.64 ± 0.02	21.85 ± 0.15	0.10 ± 0.01	–	1.87 ± 0.00	11.91 ± 0.87	0.14 ± 0.02	–	1.07 ± 0.09	2.90 ± 0.93
CzDGAT1A	1.09 ± 0.05	1.37 ± 0.05	11.51 ± 0.14	1.07 ± 0.12	5.51 ± 0.77	1.05 ± 0.02	7.18 ± 0.16	1.06 ± 0.04	3.05 ± 0.06	1.19 ± 0.02	22.39 ± 1.37	1.33 ± 0.01	4.68 ± 0.31
CzDGAT1B	0.24 ± 0.03	0.73 ± 0.01	12.20 ± 0.06	1.09 ± 0.01	12.02 ± 0.19	0.28 ± 0.01	19.91 ± 0.94	1.40 ± 0.04	15.16 ± 0.25	0.92 ± 0.01	14.15 ± 0.78	1.38 ± 0.01	3.86 ± 0.17
CzDGTT1	0.11 ± 0.02	0.36 ± 0.14	15.60 ± 3.23	0.92 ± 0.01	14.04 ± 0.00	0.24 ± 0.05	26.79 ± 8.79	1.08 ± 0.05	9.24 ± 0.25	0.19 ± 0.04	–	1.12 ± 0.08	4.83 ± 0.14
CzDGTT2	0.13 ± 0.04	0.13 ± 0.00	–	0.99 ± 0.01	16.37 ± 0.02	0.15 ± 0.00	–	1.24 ± 0.03	10.29 ± 0.42	0.50 ± 0.07	22.20 ± 0.79	1.21 ± 0.02	4.84 ± 0.05
CzDGTT3	0.18 ± 0.02	0.31 ± 0.03	–	1.64 ± 0.04	24.44 ± 0.44	0.29 ± 0.00	–	2.22 ± 0.02	22.19 ± 0.24	0.24 ± 0.01	–	1.02 ± 0.00	2.62 ± 0.08
CzDGTT4	0.10 ± 0.01	0.09 ± 0.01	–	1.13 ± 0.03	7.86 ± 0.34	0.20 ± 0.01	–	1.51 ± 0.09	23.49 ± 0.44	0.16 ± 0.03	–	1.20 ± 0.02	1.91 ± 0.19
CzDGTT5	0.27 ± 0.02	0.43 ± 0.03	11.99 ± 0.25	1.03 ± 0.02	14.23 ± 0.13	0.27 ± 0.00	9.83 ± 0.11	1.30 ± 0.00	14.84 ± 0.30	0.35 ± 0.04	4.08 ± 0.23	1.21 ± 0.01	1.49 ± 0.38
CzDGTT6	0.13 ± 0.02	0.08 ± 0.01	–	1.23 ± 0.04	16.09 ± 0.35	0.43 ± 0.03	–	1.68 ± 0.06	21.30 ± 0.04	0.18 ± 0.01	–	0.98 ± 0.00	0.86 ± 0.09
CzDGTT7	0.19 ± 0.06	0.26 ± 0.01	–	1.13 ± 0.04	14.41 ± 0.02	0.15 ± 0.01	–	1.27 ± 0.06	8.71 ± 0.35	0.14 ± 0.01	–	1.00 ± 0.06	0.98 ± 0.34
CzDGTT8	0.12 ± 0.01	0.19 ± 0.04	–	1.42 ± 0.06	24.91 ± 0.12	0.22 ± 0.02	–	1.79 ± 0.03	23.01 ± 0.48	0.16 ± 0.02	–	1.20 ± 0.14	0.50 ± 0.07

The data are expressed as mean ± SD (*n *= 3). *PL* polar lipids, *FFAs* free fatty acids, *–* under detectable level

### Acyl-CoA substrate specificity of CzDGAT1A and CzDGTT5

**Fig. 3:**
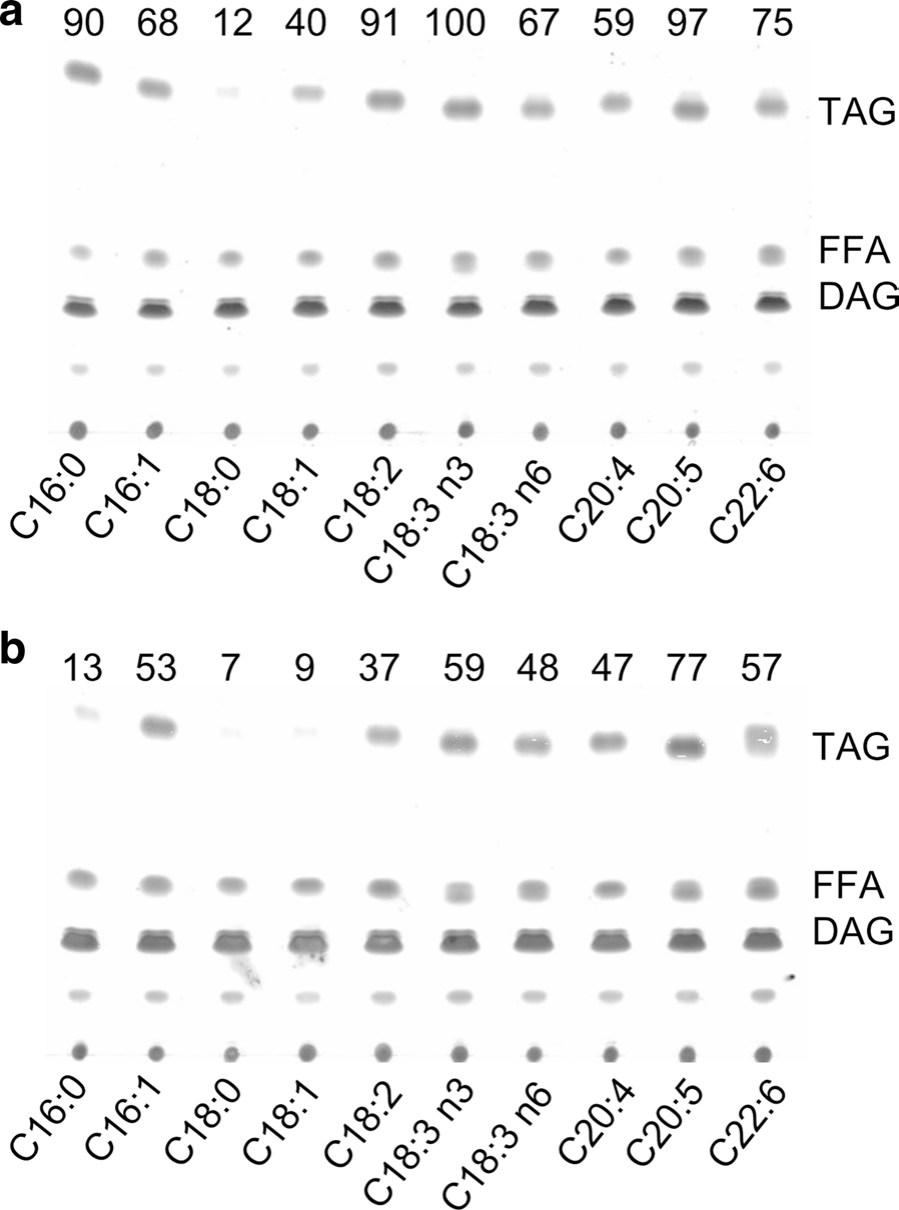
The in vitro substrate specificities of CzDGAT1A and CzDGTT5 for acyl-CoAs. Lipids resulting from in vitro enzymatic reactions of CzDGAT1A (**a**) and CzDGTT5 (**b**) with various acyl CoAs were analyzed by TLC. C18:1/C18:1-DAG was used as the acyl-CoA acceptor. The numbers above each panel indicate the relative TAG values, normalized to that obtained for CzDGAT1A toward C18:3*n*3 (set as 100) by using the Image J software

Although the exogenous FFA feeding experiments provided implications into the acyl-CoA substrate specificity of *C. zofingiensis* DGATs, solid experimental evidences are lacking, which can be addressed by in vitro assay. We have recently developed a non-radiolabeled DGAT in vitro assay [40], which allows for the measurement of activity and substrate specificity of DGAT toward a wide range of acyl-CoAs and DAGs, and has been well applied to DGATs from *C. reinhardtii* [25] and *N. oceanica* [27, 28]. Given that CzDGAT1A and CzDGTT5 showed the strongest acyltransferase activity (Fig. 1), here we focused on these two enzymes for in vitro assay. As the *sn*-2 position of TAG from *C. zofingiensis* contains mainly 18-carbon acyls [32], the eukaryotic C18:1/C18:1-DAG was used as the acyl acceptor for acyl-CoA substrate specificity assay. Overall, CzDGAT1A showed greater activity than CzDGTT5 regardless of acyl-CoAs (Fig. 3), consistent with the functional complementation results that CzDGAT1A made the yeast produce more TAG than CzDGTT5 did (Fig. 2). Specifically, for C16-CoAs, CzDGAT1A preferred the saturated one (C16:0) over the unsaturated one (C16:1); while for C18-CoAs, CzDGAT1A preferred polyunsaturated ones (C18:2 and C18:3), followed by the monounsaturated one (C18:1) and saturated one (C18:0) (Fig. 3a). CzDGTT5 also preferred the polyunsaturated ones of C18-CoAs (Fig. 3b). Nevertheless, for C16-CoAs, CzDGTT5 had a considerable higher activity on C16:1-CoA than on C16:0-CoA (Fig. 3b), distinct from the preference of CzDGAT1A (Fig. 3a). When the double bond number was the same, CzDGAT1A and CzDGTT5 both preferred shorter-chain acyl-CoAs, as indicated by the results that C16:0 and C16:1 led to more TAG than C18:0 and C18:1, respectively (Fig. 3). Intriguingly, both CzDGAT1A and CzDGTT5 had activity on the long-chain polyunsaturated acyl-CoAs of C20:4, C20:5 and C22:6 (Fig. 3), though these are not present in *C. zofingiensis*.

### DAG substrate specificity of CzDGAT1A and CzDGTT5

**Fig. 4:**
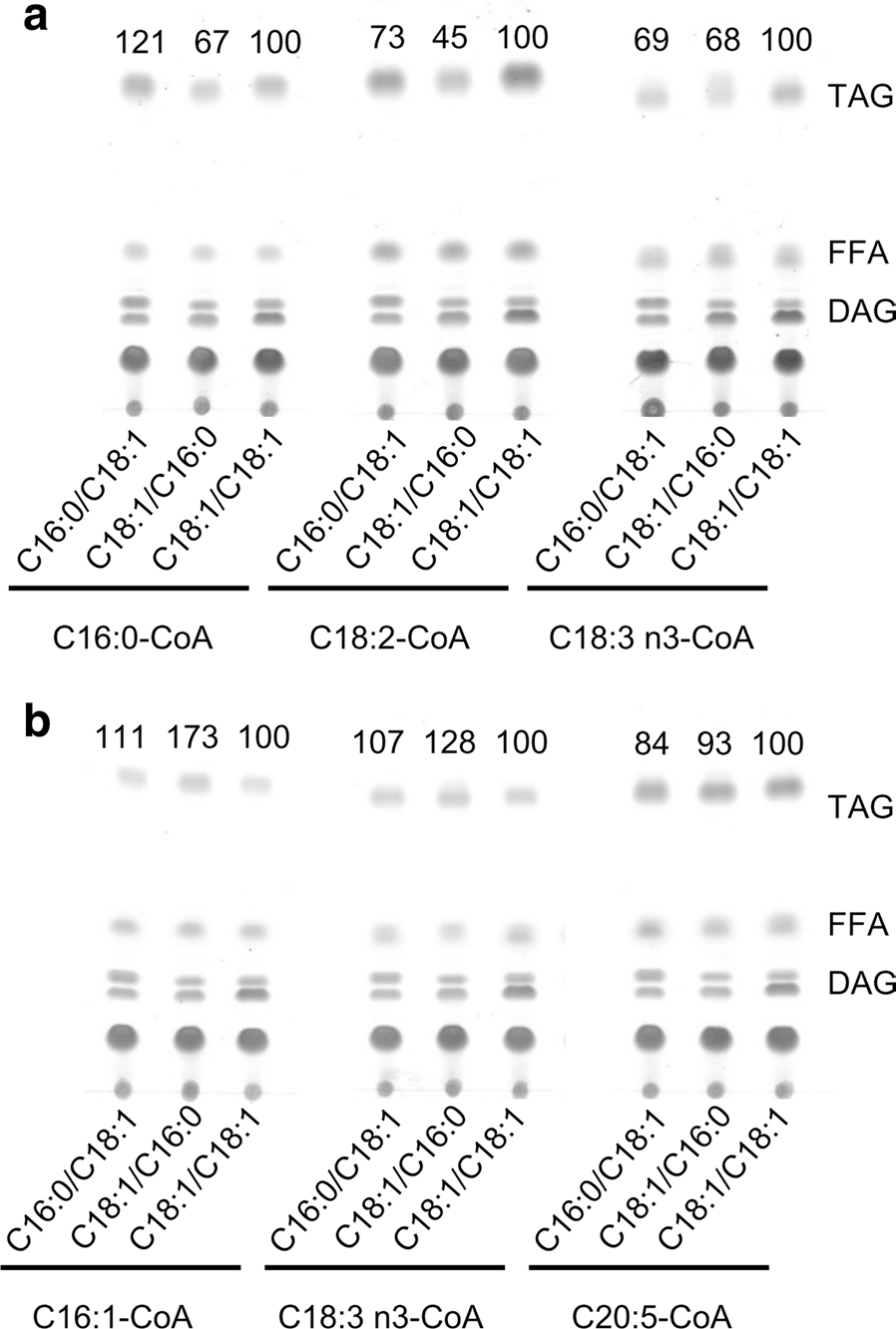
The in vitro substrate specificities of CzDGAT1A and CzDGTT5 for DAGs. Lipids resulting from in vitro enzymatic reactions of CzDGAT1A (**a**) and CzDGTT5 (**b**) with various DAGs were analyzed by TLC. The numbers above each panel indicate the relative TAG values, normalized to those obtained when C18:1/C18:1-DAG was used as the acyl acceptor (set as 100) by the Image J software

We further investigated the preference of CzDGAT1A and CzDGTT5 for various DAGs, including one prokaryotic DAG (C18:1/C16:0) and two eukaryotic DAGs (C16:0/C18:1 and C18:1/C18:1). Obviously, regardless of acyl-CoAs (C16:0-CoA, C18:2-CoA and C18:3*n*3-CoA) used as the acyl donor, CzDGAT1A exhibited greater activity towards C16:0/C18:1- and C18:1/C18:1-DAGs than C18:1/C16:0-DAG (Fig. 4a), indicative of its preference on eukaryotic DAG over prokaryotic one for TAG synthesis. By contrast, CzDGTT5 preferred prokaryotic DAG for TAG formation, as evidenced by the fact that more TAG was produced with C18:1/C16:0-DAG than with C16:0/C18:1- and C18:1/C18:1-DAGs, particularly when C16:1-CoA or C18:3*n*3-CoA was used as the acyl donor (Fig. 4b).

### Subcellular localization of CzDGAT1A and CzDGTT5 in tobacco leaves

**Fig. 5:**
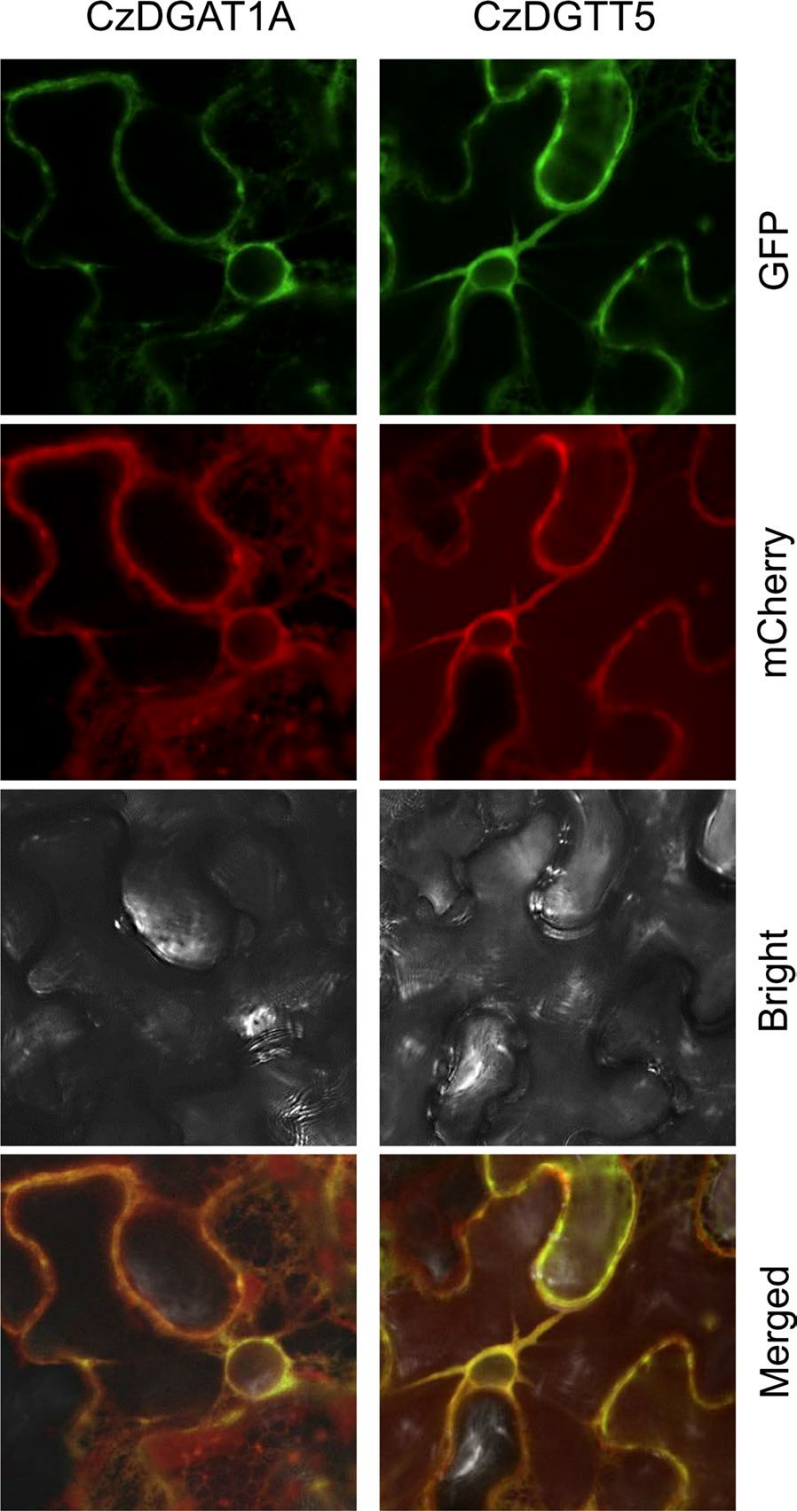
Subcellular localization of CzDGAT1A and CzDGTT5 in tobacco lower epidermal leaf cells. DGAT::GFP was co-localized with a mCherry-tagged endoplasmic reticulum (ER) marker

Type I and type II DGATs are membrane-bound proteins and are thought to be ER-localized in higher plants [41]. The compartmentalization of DGAT in algae, however, has been rarely touched and appears to be ambiguous [25, 27, 29, 42]. To confirm the localization of CzDGAT1A and CzDGTT5, a C-terminally tagged GFP fusion was employed. As such a system is so far not available in *C. zofingiensis*, we chose tobacco leaf as the expression host: Cz*DGAT1A* and Cz*DGTT5* were each transiently co-expressed in lower epidermal leaf cells with the mCherry-tagged ER marker ER-rk CD3-959 [43]. Clearly, the GFP signal was overlaid well with the ER marker for both CzDGAT1A and CzDGTT5 (Fig. 5), suggesting their localization at ER.

### Possible transcription factors for *CzDGAT1A* and *CzDGTT5*

Transcription factors (TFs) represent a group of regulators controlling their target gene expression at the transcriptional level through binding certain upstream elements. An increasing number of TFs that are involved in lipid synthesis regulation have been predicted and/or identified in algae, including MYB and bZIP [23, 44–46]. To screen the possible TFs for *CzDGAT1A* and *CzDGTT5*, eight MYBs and six bZIPs were evaluated. Yeast one-hybrid assay indicated that MYB1 (Cz02g00230) bound with *Cz*DGTT5 promoter, while bZIP3 (Cz15g21170) bound both with *CzDGAT1A* and *CzDGTT5* promoters (Additional file 1: Figure S5). Analysis of the promoter sequences by the software PlantPAN 2.0 [47] predicted many potential binding sites of MYB and bZIP (Additional file 3: Data S1), which were mapped at the promoter regions of *CzDGAT1A* and *CzDGTT5*, with their 5′ neighboring genes (Cz06g05020 and Cz09g27300) shown (Additional file 1: Figures S6, S7). Similar to *CzDGAT1A* and *CzDGTT5*, MYB1 and bZIP3 were up-regulated by ND at the transcriptional level; by contrast, Cz06g05020 and Cz09g27300 showed little changes upon ND (Additional file 1: Figure S8), demonstrating the expression correlation between TFs with *CzDGAT1A* and *CzDGTT5* rather than with their neighboring genes. In this context, MYB1 and bZIP3 are probably the TFs regulating *CzDGAT1A* and *CzDGTT5*.

### Engineering potential *C. zofingiensis DGAT*s for TAG modulation in yeast and algae

**Fig. 6:**
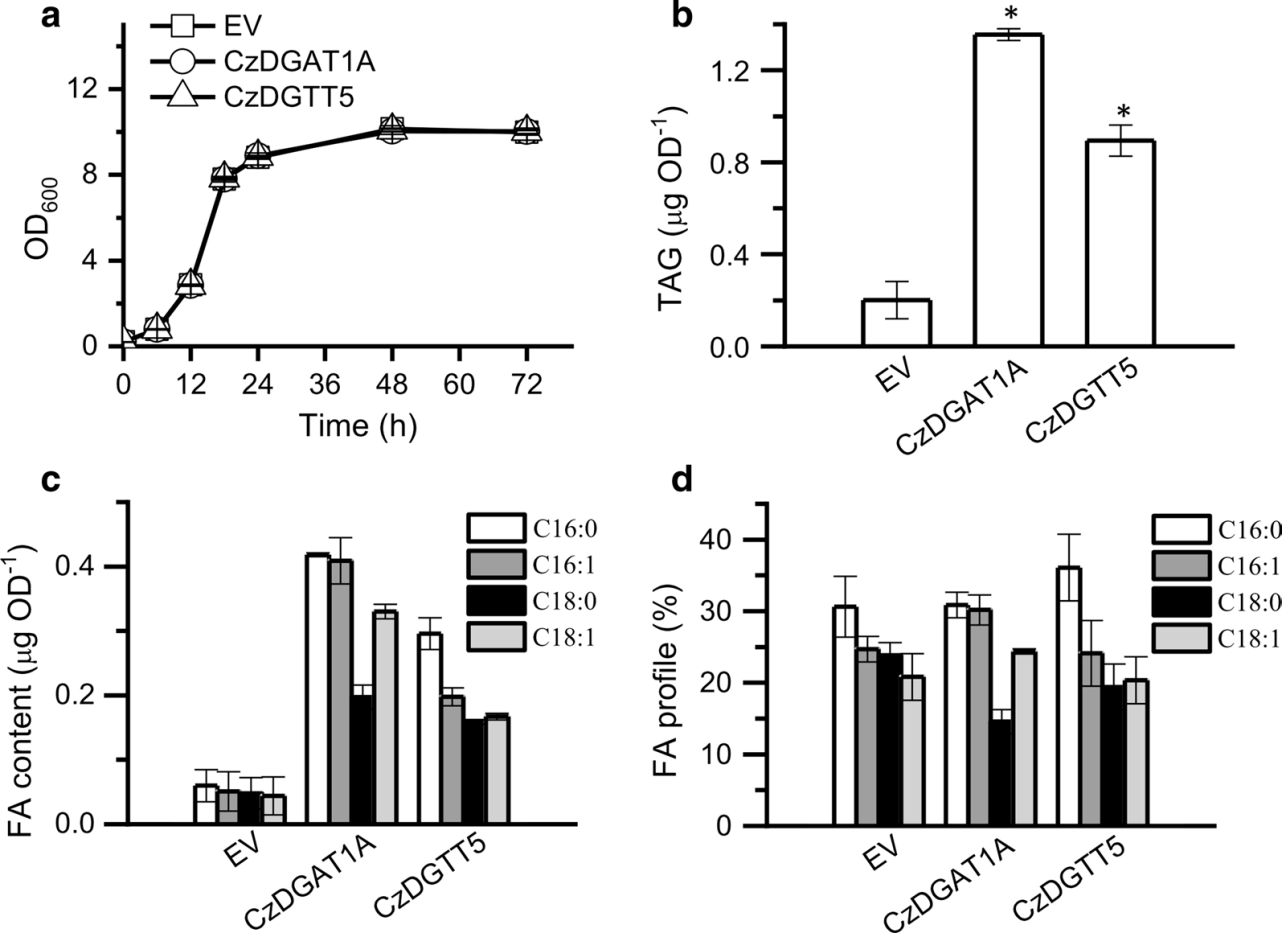
Overexpression of Cz*DGAT1A* and Cz*DGTT5* each in the yeast strain *S. cerevisiae* INVSc1. **a** Growth curve. **b** TAG content. **c** Fatty acid content of TAG. **d** Fatty acid percentage of TAG. The data were obtained from 48-h cultures and are expressed as mean ± SD (*n* = 3). Asterisks indicate the significant difference compared to EV (*t*-test, *P* < 0.01)

To see if *C. zofingiensis DGAT*s have the potential to improve TAG synthesis, *CzDGAT1A* and *CzDGTT5* were individually introduced into the yeast strain INVSC1 to examine their impact on TAG synthesis. The overexpression of *CzDGAT1A* and *CzDGTT5* each had no effect on the growth of yeast cells (Fig. 6a), but promoted TAG accumulation considerably (Fig. 6b). Accordingly, the greater TAG yield was achieved in *CzDGAT1A*- and *CzDGTT5*-expressing strains, 6.7- and 4.5-fold higher than the EV control, respectively. The FA composition was only slightly affected by the expression of the two genes (Fig. 6c).

**Fig. 7:**
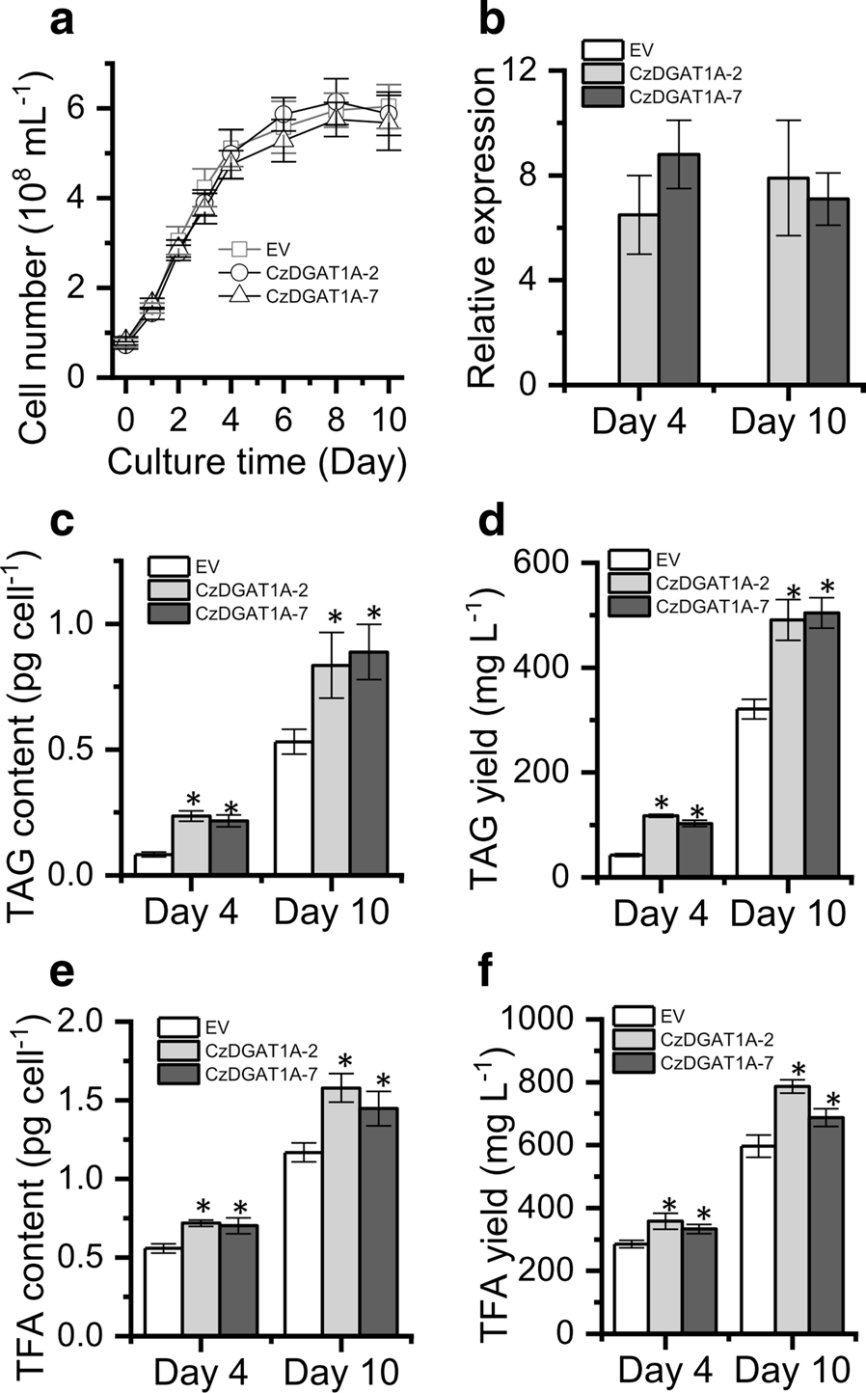
Overexpression of Cz*DGAT1A* in the alga *N. oceanica*. **a**–**f** Cell number (**a**), *DGAT1A* expression (normalized to endogenous β-actin gene) (**b**), TAG content (**c**), TAG yield (**d**), TFA content (**e**) and TFA yield (**f**) between Cz*DGAT1A*-overexpressing lines and the EV. The data were obtained from 48-h cultures and are expressed as mean ± SD (*n* = 3). Asterisks indicate the significant difference compared to EV (*t*-test, *P* < 0.01)

We also introduced *CzDGAT1A* into the oleaginous alga *N. oceanica* to assess its effect on TAG production. No significant difference was observed in cell growth between the transgenic lines and EV control (Fig. 7a). *CzDGAT1A* expression in *N. oceanica*, confirmed by quantitative real-time PCR (Fig. 7b), resulted in a considerable increase (~ 2.8-fold) in TAG level in the linear growth stage (day 4) (Fig. 7c). The TAG augmentation was also observed in the stationary growth stage (day 10), though the increase extent was less than in the linear growth stage (Fig. 7c). Accordingly, the *CzDGAT1A*-overexpressing lines gave a high TAG yield, 58% greater than that of the EV at the end of culture period (Fig. 7d). The overexpression of *CzDGAT1A* also promoted TFA content (Fig. 7e) and yield (Fig. 7f). These results indicate that *CzDGAT1A* has the engineering potential in improving the production of lipids particular TAG.

## Discussion

*Chlorella zofingiensis* represents a leading algal candidate of industrial potential because of its ability to synthesize both TAG and astaxanthin, which can be induced by many factors such as nitrogen deprivation, sulfur deprivation, and high light [5, 10, 32]. TAG content and its fatty acid composition, two important parameters for evaluating the potential of an alga for biodiesel production, can be modulated by the expression of *DGAT*s [27–29, 48], as DGAT is believed to catalyze the last committed step of TAG assembly and play a key role in controlling TAG accumulation. *C. zofingiensis* was predicted to possess a total of eleven putative DGAT-encoding genes [31]. However, we demonstrated that two of them, Cz03g14080 and Cz09g23020, had identical sequence and should be at the same locus of the genome (Additional file 1: Figure S1), indicating the presence of ten *DGAT*s in the alga. To characterize the roles of *C. zofingiensis* DGATs, we cloned and identified the ten *DGAT* genes with confirmed full-length coding sequence (Additional file 1: Figure S2). Expression in the TAG-deficient yeast strain H1246, a commonly used system for DGAT functional complementation [36], confirmed that seven of the ten *DGAT* genes are functional, despite the large difference in enzymatic activity (Fig. 2). The functional failure of the other *DGAT* genes may be caused by the absence of certain fatty acids in yeast such as C18:2 and C18:3, which are present in *C. zofingiensis*. This holds true, at least for CzDGTT1, because the feeding of the two free fatty acids each made the CzDGTT1-expressing yeast produce more TAG enriched with the fed fatty acids (Table 2). It is also possible that some of these genes may encode not a real DGAT but other types of transferase, which cannot be distinguished based only on the sequence data. Among the green algae, *C. reinhardtii* is the best studied one for DGAT characterization: CrDGTT1 through CrDGTT3 are functional while CrDGTT4 is not [24–26]. Considering the close relation of *C. zofingiensis* DGATs (CzDGTT1, CzDGTT2, CzDGTT4, and CzDGTT5) to *C. reinhardtii* ones (CrDGTT1 through CrDGTT4) (Additional file 1: Figure S3), it appears that the functionality for DGATs is phylogenetically conserved. It has been suggested in *H. pluvialis* that DGATs may be involved in the esterification of astaxanthin [33]. To examine if *C. zofingiensis* DGATs catalyze the formation of astaxanthin ester, we expressed all ten *DGAT*s in a reconstructed astaxanthin-producing yeast strain [49]. Nevertheless, no astaxanthin ester was detected, indicating the null function of *C. zofingiensis* DGATs in astaxanthin esterification.

The functional complementation experiment in yeast tells which algal DGATs are functional (Fig. 2) but not their substrate specificity, which can be addressed by in vitro assay. We have recently developed a nonradiolabeled in vitro DGAT assay featured by the use of widely available and regular acyl-CoAs and DAGs [40], which has been successfully applied to examine both type I and type II DGATs from algae [25, 27, 28]. By means of this assay, CzDGAT1A and CzDGTT5, which showed the highest activity in H1246 (Fig. 2), were evaluated with a wide range of acyl-CoAs present in *C. zofingiensis*. There have been many reports about the functional characterization of DGATs from algae, but mainly restricted to the complementation assay in TAG-deficient yeast [23, 24, 39, 50–52]. Only a few studies conducted the in vitro characterization of algal type I DGATs [27] or type II DGATs [25–28]. Our work here represents the first effort to elucidate the in vitro substrate preference of both algal type I and type II DGATs, which revealed the overlapping yet distinctive preference of CzDGAT1A and CzDGTT5 for acyl-CoAs, e.g., CzDGTT5 had weak activity on C16:0-CoA and C18:1-CoA while CzDGAT1A had strong activity on these two acyl-CoAs (Fig. 3). Moreover, CzDGAT1A and CzDGTT5 accepted both prokaryotic and eukaryotic DAGs for TAG assembly, but CzDGAT1A preferred eukaryotic DAGs while CzDGTT5 preferred prokaryotic ones (Fig. 4). These results suggest the functional complementarity of type I and type II DGATs for TAG synthesis in *C. zofingiensis*. Unlike *N. oceanica* DGAT1A that has activity only on saturated and monounsaturated acyl-CoAs [27], CzDGAT1A is active also on the polyunsaturated acyl-CoAs (Fig. 3a). Similarly, CzDGTT5 and its close homolog, CrDGTT3 (Additional file 1: Figure S3), exhibit distinct preference for acyl-CoAs (Fig. 3b; [25]). *N. oceanica* DGAT2A, which is thought to originate from green algae [22] and somewhat close to CzDGTT5 (Additional file 1: Figure S3), also differs from CzDGTT5 in the preference for acyl-CoAs [28]. These differences indicate the diversification of DGATs regarding substrate preference and activity during the evolution.

In addition to the de novo synthesized fatty acids (C16:0, C18:0 and C18:1), *C. zofingiensis* TAG contains membrane lipids-deprived polyunsaturated fatty acids (Table 1), indicative of the contribution of membrane lipids to TAG. This is further supported by the fact that TAG accumulates at the expense of membrane lipids, particularly glycolipids [5]. The phenomenon appears to be universal as it occurs in many other algae including *Chlamydomonas* [25], *Chlorella* [53], *Nannochloropsis* [27], and *Phaeodactylum* [54]. In higher plants, it is thought that glycerolipids with a C18 acyl group in the *sn*-2 position are assembled via the eukaryotic pathway, whereas those with a C16 acyl group in *sn*-2 are from the prokaryotic pathway [55]. If this holds true for algae, *Chlamydomonas* and *Nannochloropsis* may involve predominantly the prokaryotic pathway for TAG assembly, as their TAG *sn*-2 position consists mostly of C16 acyls [27, 56, 57]. By contrast, *C. zofingiensis* TAG contains predominantly C18 acyls in its *sn*-2 position [32], and is, thus, likely contributed mainly by the eukaryotic pathway. Besides, C16:0 and C18:1 are the most abundant fatty acids in the *sn*-1/3 positions of *C. zofingiensis* TAG [32]. In this context, considering that CzDGAT1A prefers eukaryotic DAGs while CzDGTT5 prefers prokaryotic DAGs and CzDGAT1A has much higher activity than CzDGTT5 on the acyl-CoAs of C16:0 and C18:1 (Figs. 3 and 4), CzDGAT1A is likely to play a more important role in contributing to TAG accumulation in *C. zofingiensis*. Nevertheless, we could not rule out the possibility that the heterologous and in vitro results may not reflect the in vivo function of *C. zofingiensis* DGATs, although our previous studies have demonstrated the consistence among heterologous, in vitro and in vivo results for DGATs in other algae [25, 28]. In vivo characterization of *zofingiensis* DGATs will provide an additional layer of evidence, which relies on future investigation when the rational manipulation of the alga for gene overexpression and/or suppression is available.

Because of the blooms in algal lipids for fuels and value-added products, the characterization of DGATs has attracted increasing interests, from the model alga *C. reinhardtii* to industrially relevant oleaginous algae such as *Nannochloropsis* and *Chlorella*. These studies provide gene sources used not only in algae but also in higher plants for improving oil content and the fatty acid composition [26–29, 51, 58, 59]. It is worth noting that CrDGTT1 has a much higher activity than other *C. reinhardtii* DGATs and *N. oceanica* DGATs [25, 27, 28]. In the present study, CzDGAT1A and CzDGTT5, particularly CzDGAT1A, showed a considerable higher activity than *C. reinhardtii* DGTT1 (Fig. 2); besides, heterologous expression of *CzDGAT1A* promoted TAG production considerably in either yeast (Fig. 6) or the alga *N. oceanica* (Fig. 7). These data together indicate the superior potential of CzDGAT1A for oil modulation in oleaginous algae and maybe in oil crops as well. TAG accumulation involves a serial of collaborative steps and manipulating a single gene, e.g., DGAT mentioned above, cannot achieve a satisfactory need. TFs involved in TAG metabolism are of particular interest, because they master a set of genes for TAG synthesis and utilizing them as the engineering target could bypass the manipulation of multiple genes and achieve better performance in promoting TAG production [46, 60]. Moreover, the manipulation of TF has the potential to turn on TAG accumulation under non-stress conditions, thereby avoiding the stress-associated growth compromise, which would be of great benefit to biotechnological applications. It has been demonstrated that overexpression of certain TFs in algae led to enhanced lipid synthesis to various extents [61–64]. In *C. zofingiensis*, bZIP3, which probably regulates *CzDGAT1A* and *CzDGTT5* (Additional file 1: Figure S6), represents an interesting target of engineering for increasing TAG production and is worth of trying in the future studies. The development of overexpression platforms for *C. zofingiensis* is underway in our laboratory, which will pave the way toward manipulating this alga for trait improvements.

## Conclusions

**Fig. 8:**
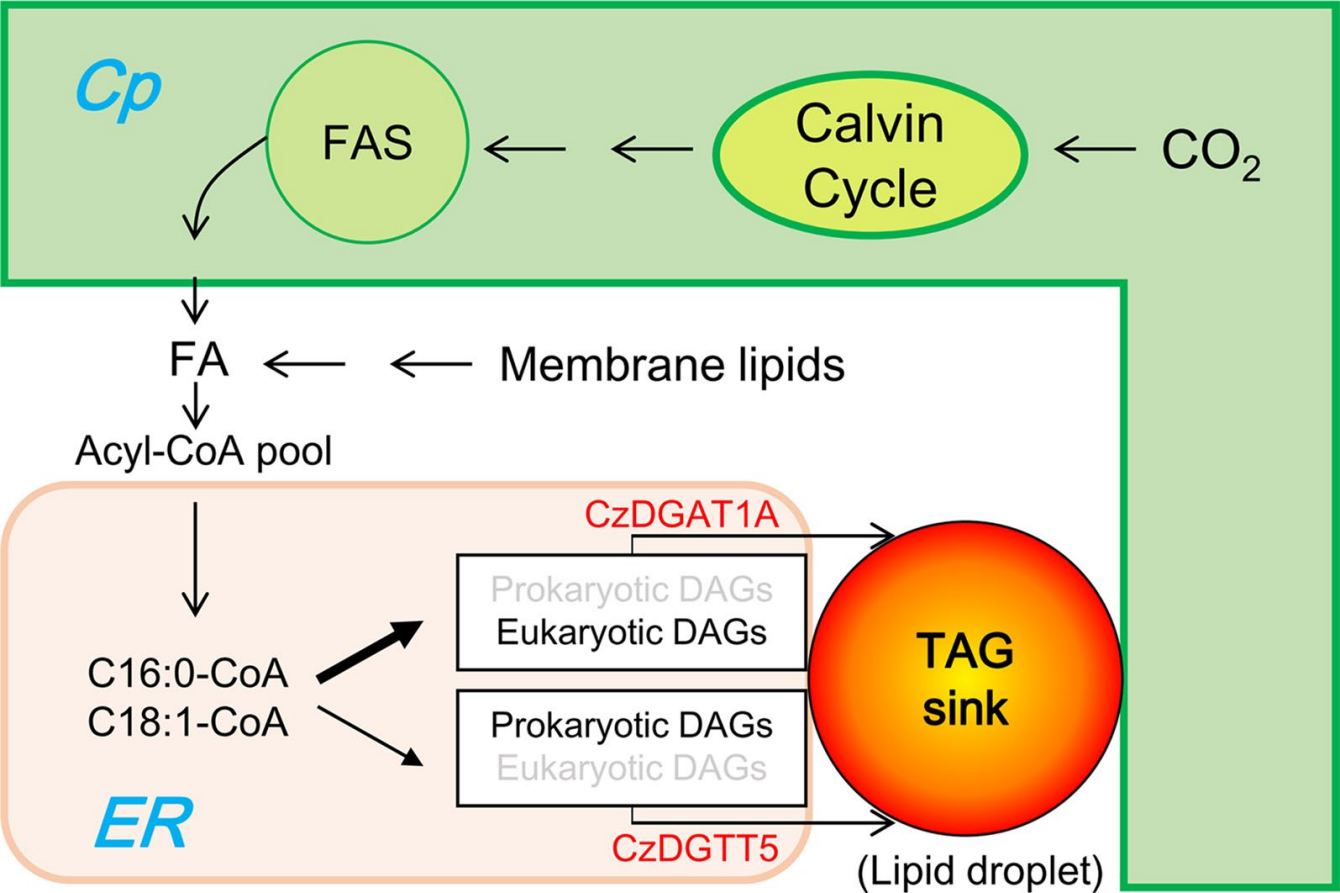
A hypothesized working model illustrating the localization and possible roles of CzDGAT1A and CzDGTT5 in TAG biosynthesis in *C. zofingiensis*. The de novo synthesized fatty acids, together with those released from membrane polar lipids, constitute the acyl-CoA pool. In response to ND, CzDGAT1A and CzDGTT5 are up-regulated considerably and localize at ER. DGAT1A utilizes more C16:0- and C18:1-CoAs than CzDGTT5 and prefers eukaryotic DAGs as the acyl acceptor for TAG assembly, while CzDGTT5 prefers prokaryotic DAGs for TAG assembly. The prokaryotic DAGs may be translocated from chloroplast to the ER by diffusion along the delimiting monolayer of lipid droplet, which has contact sites with both chloroplast and ER. Not all intermediates or reactions were displayed. *ER* endoplasmic reticulum, *Cp* chloroplast, *FA* fatty acids

Here, we performed an in-depth characterization of *C. zofingiensis* type I and type II DGATs by systematically integrating gene cloning and bioinformatics analysis, functional complementation in TAG-deficient yeast, in vitro DGAT assay for substrate specificity, subcellular localization, yeast one-hybrid assay for identifying TFs, and overexpression in different hosts for oil modulation. A working model was proposed for the role of CzDGAT1A and CzDGTT5 in TAG biosynthesis in the oleaginous alga *C. zofingiensis* (Fig. 8). The fatty acyls de novo synthesized and/or recycled from the turnover of membrane lipids constitute the acyl-CoA pool and enter Kennedy pathway for the synthesis of both prokaryotic and eukaryotic DAGs. CzDGAT1A and CzDGTT5, the two representative type I and type II DGATs, are up-regulated in response to ND and reside at ER. Differing from CzDGTT5 that uses mainly prokaryotic DAGs and has weak activity on C16:0- and C18:1-CoAs, DGAT1A prefers eukaryotic DAGs with strong activity on C16:0- and C18:1-CoAs, thus contributing more to the synthesis of TAG in *C. zofingiensis*. The prokaryotic DAGs may be translocated from chloroplast to the ER by diffusion along the lipid droplet-delimiting monolayer as hypothesized by Goodson et al. [65], and accessed by CzDGAT1A and CzDGTT5 for TAG assembly. Moreover, overexpression of *CzDGAT1A* and *CzDGTT5* genes promoted TAG production in different hosts including yeast and oleaginous alga. Taken together, our study represents a pioneering work on the characterization of both type I and type II DGATs from algae, which not only helps to better understand the mechanism of TAG biosynthesis in *C. zofingiensis*, in which CzDGAT1A and CzDGTT5 have functional complementarity and likely work in collaboration at ER for TAG assembly, but also provides insights into future genetic engineering of the alga by manipulating rate-limiting enzymes such as CzDGAT1A or TFs such as bZIP3 to modulate oil production.

## Methods

### Algal strains and culture conditions

*Chlorella zofingiensis* (ATCC 30412) was obtained from the American Type Culture Collection (ATCC, Rockville, MD, USA) and maintained and cultured in the Kuhl medium as described by our previous study [32]. Briefly, 10 mL of liquid Kuhl medium was inoculated with cells from agar plates and the alga was grown aerobically in flasks at 25 °C for 6 days with orbital shaking at 150 rpm and illuminated with continuous light of 30 µE m^−2^ s^−1^. The cells were then inoculated at 10% (v/v) into 250-mL columns (3-cm diameter) provided with constant illumination of 70 µE m^−2^ s^−1^ and aeration of 1.5% CO_2_ enriched air. For nitrogen deprivation (ND) experiments, the cells grown to late exponential phase were harvested, washed with nitrogen-deficient medium twice and suspended in this medium (cell density, 0.5 g L^−1^), and transferred to new 250-mL columns for growth. The culture conditions are the same as mentioned above.

*Nannochloropsis oceanica* IMET1 was from Institute of Marine and Environmental Technology, University Systems of Maryland. It was maintained at 16 °C on an agar plate of the modified F/2 medium (100 mg L^−1^ N and 4.5 mg L^−1^ P) containing 20 g L^−1^ sea salt and grown in 250-mL columns (3-cm diameter) provided with constant illumination of 70 μE m^−2^ s^−1^ and aeration of 1.5% CO_2_ enriched air [27].

### Cloning and bioinformatics analysis of *C. zofingiensis DGAT*s

To obtain the full-length coding sequence of *C. zofingiensis DGAT*s, their transcription start sites were first determined by 5′ rapid amplification of cDNA ends (RACE)-PCR using the SMARTer RACE 5′/3′ Kit (Clontech, CA, USA) and the 5′ GSPs (Gene-Specific Primers) primers (Additional file 1: Table S1). All of the amplified fragments were sequenced. Then, primer pairs were used to amplify the full-length coding sequences (Additional file 1: Figure S2): the forward primer was designed to locate right upstream the start codon based on the confirmed 5′ UTR sequence in our study, and the reverse primer was designed to locate right downstream of the stop codon based on the gene model from Roth et al. [31]. Using cDNA as the template, the primer pairs of all *C. zofingiensis DGAT* genes except *CzDGTT7* gave PCR products. Analyzing its genomic sequence predicted the presence of an intron right after the gene based on the gene model from Roth et al. [31]; thus, an additional reverse primer was designed (Additional file 1: Figure S2). The full-length coding sequences of *DGAT*s were verified by sequencing, which were deposited into NCBI Genbank with accession numbers (Additional file 2: Table S1).

Sequence alignment of DGAT polypeptides from various organisms was conduct using ClustalX2.1 (http://www.clustal.org/clustal2/) and the phylogenetic tree was generated using MEGA6 [34]. Conserved domains of *C. zofingiensis* DGAT proteins were detected through the NCBI Conserved Domains Search (https://www.ncbi.nlm.nih.gov/Structure/cdd/wrpsb.cgi). Transmembrane helices were predicted using TMHMM 2.0 (http://www.cbs.dtu.dk/services/TMHMM/). Subcellular location prediction was performed using PredAlgo program, a multi-subcellular localization prediction tool dedicated to algae (http://giavap-genomes.ibpc.fr/predalgo), TargetP (http://www.cbs.dtu.dk/services/TargetP/), and ChloroP (http://www.cbs.dtu.dk/services/ChloroP/). The prediction of potential binding sites of MYB and bZIP at the promoter regions of *CzDGAT1A* and *CzDGTT5* was performed with the software PlantPAN 2.0 [47].

### RNA isolation and quantitative real-time PCR

RNA extraction from algae samples and removal of contaminated DNA were conducted using the plant RNA extraction kit (TaKaRa, Japan) according to the manufacturer’s instructions. The total RNA concentration was determined by NannoDrop 2000c (Thermo Scientific, DE, USA) and the quality was checked by electrophoresis. The cDNA synthesis and quantitative real-time PCR were performed as described by Liu et al. [25] using a 7500 Fast Real-Time PCR System (Applied Biosystems, Waltham, MA, USA) with SYBR^®^ Premix Ex Taq™ II (Tli RNase H Plus) (TaKaRa). Primer sequences were used for quantitative real-time PCR, see Mao et al. [32]. The mRNA expression level was normalized using the actin gene as the internal control.

### Functional complementation of *C. zofingiensis DGAT*s in the TAG-deficient yeast H1246

The type I and type II *DGAT* genes from *C. zofingiensis* including *CzDGAT1A, CzDGAT1B*, and *CzDGTT1* through *CzDGTT8* (Additional file 1: Table S1) were PCR amplified using cDNA as template and cloned into the yeast expression vector pYES2-CT (Invitrogen, CA, USA). PCR primers for cloning are listed in Additional file 1: Table S3. After confirmation by restriction enzyme digestion and sequencing, the recombinant pYES2-DGAT plasmids were each transformed into the *Saccharomyces cerevisiae* TAG-deficient quadruple mutant strain H1246 [36] using S.c. EasyComp Transformation Kit (Invitrogen). Colony PCR was used to verify the presence of the plasmids in the transformants. H1246 cells carrying the empty vector pYES2-CT (EV control) and pYES2-CrDGTT1 (containing a type II *DGAT* gene from *C. reinhardtii*) were from Liu et al. [25]. The expression of *DGAT*s was induced by 2% (w/v) galactose in SD/-ura medium [40]. When necessary, free fatty acids were fed to yeast cultures as described by Siloto et al. [37], with supplementation of linoleic acid (C18:2), α-linolenic acid (C18:3n3), and eicosapentaenoic acid (C20:5n3) at a concentration of 125 µM upon galactose induction.

After induction with galactose for 2 days, H1246 cells were harvested for lipid extraction and analysis (see below section) and staining with BODIPY 493/503 (Invitrogen), a neutral lipid-specific fluorescent dye. The lipid droplets in the cells stained with BODIPY (at a working concentration of 10 μg mL^−1^) were visualized under fluorescence microscope (Olympus, Japan).

### In vitro enzymatic assay for *C. zofingiensis* DGATs

The H1244 transformants bearing *CzDGAT1A* and *CzDGTT5* were each grown in liquid SD/-ura medium containing 2% (w/v) galactose for 18 h at 30 °C, and then harvested for microsome preparation using a French pressure cell (Spectronics Instruments, NY, USA). The detailed procedures were described by Liu et al. [40]. The resulting microsomal membrane pellets were resuspended in microsome storage buffer (50 mM Tris–HCl, pH 7.5, 10% glycerol) to give a protein concentration of 10 µg µL^−1^ for immediate use or stored at − 80 °C.

The in vitro DGAT assay was conducted according to our previously described procedures [40]. The acyl-CoAs tested included palmitoyl-CoA (C16:0-CoA), hexadecenoyl-CoA (C16:1-CoA), stearoyl-CoA (C18:0-CoA), oleoyl-CoA (C18:1-CoA), linoleoyl-CoA (C18:2-CoA), α-linolenoyl-CoA (C18:3n3-CoA), γ-linolenoyl-CoA (C18:3n6-CoA), arachidonyl-CoA (C20:4-CoA), eicosapentaenoyl-CoA (C20:5-CoA), and docosahexaenoyl-CoA (C22:6-CoA). The DAGs tested were C18:1/C16:0-, C16:0/C18:1-, and C18:1/C18:1-DAGs. C16:0/C18:1- and C18:1/C18:1-DAGs were purchased from Larodan Fine Chemicals (Malmo, Sweden), whereas C18:1/C16:0-DAG was prepared by partial digestion of C18:1/C16:0/C18:1-TAG (Larodan Fine Chemicals) with *Rhizopus arrhizus* lipase (Sigma-Aldrich, MO, USA) and recovery of DAG.

### Subcellular localization of *C. zofingiensis* DGATs in tobacco leaves

To examine the subcellular localization, the coding sequences of *CzDGAT1A* and *CzDGTT5* were each amplified without the stop codon and fused in frame to the upstream of GFP in the modified binary vector pCAMBIA 1300 [66]. The resulting constructs, CzDGAT1A::GFP and CzDGTT5::GFP, were each introduced into the *Agrobacterium tumefaciens* strain GV3101. *Agrobacterium*-mediated transient expression in tobacco leaves was employed for co-localization experiments [67], using the mCherry-tagged endoplasmic reticulum marker (ER-rk; CD3-959) from the Arabidopsis Biological Resource Center [43], together with CzDGAT1A::GFP or CzDGTT5::GFP. After 3 days of infiltration, cells from the lower epidermis were sampled for microscopic analyses, using a laser scanning confocal microscope (Nikon C1, Japan). The excitation wavelengths for GFP and mCherry were 488 and 543 nm, respectively, and the emission filter wavelengths were 505–530 nm for GFP and 560–615 nm for mCherry.

### Yeast one-hybrid assay

The 2-kb promoter regions (upstream of start codon) of *CzDGAT1A* and *CzDGTT5* were individually cloned into pLacZi2μ (Clontech), while the coding sequences of TFs including eight MYBs and six bZIPs were each cloned into pJG4-5 vector (Clontech). The yeast one-hybrid assay was performed by co-introducing pLacZi2μ-promoter and pJG-TF into the yeast strain EGY48, as described in the Yeast Protocols Handbook (Clontech). Transformants were grown on the synthetic dextrose plates lacking Ura and Trp, but containing X-gal (5-bromo-4-chloro-3-indolyl-b-dgalactopyranoside). The plates were incubated at 30 °C for 2 days for color development.

### Overexpression of *C. zofingiensis DGAT*s in the yeast strain INVSC1 and marine alga *N. oceanica*

The plasmids pYES2-CzDGAT1A and pYES2-CzDGTT5 mentioned above were transformed into the TAG-producing *S. cerevisiae* strain INVSC1 using S.c. EasyComp™ Transformation Kit (Invitrogen). Transformants were selected on SD/-ura plates and verified by colony PCR. The expression of *C. zofingiensis DGAT*s in yeast was induced by 2% (w/v) galactose.

For the expression of *C. zofingiensis DGAT*s in the oleaginous alga *N. oceanica*, the coding sequence of *CzDGAT1A* was amplified and cloned into the overexpression vector as described in our previous study [27]. Nuclear transformation of *N. oceanica* was performed by electroporation according to Li et al. [68]. Transformants were selected on modified F/2 plates with 2.5 μg mL^−1^ zeocin (Life Technologies, CA, USA) and verified by genomic PCR. Quantitative real-time PCR was employed to determine the overexpression level of *CzDGAT1A*.

### Analytical methods

Chlorophylls were extracted from the fresh algal cell pellets with acetone, and concentrations were calculated from the absorbance values at 645 and 663 nm according to Li et al. [69].

Lipid extracts from yeast, *C. zofingiensis*, and *N. oceanica* cells as well as the in vitro DGAT assay mixture were all performed according to our previously described procedures [25, 40].

Neutral lipids were separated on a Silica gel 60 TLC plate (EMD Chemicals, Merck, Germany) using a mixture of hexane/tert-butylmethyl ether/acetic acid (80/20/2, by volume) as the mobile phase. Then lipids were observed by spraying the TLC plate with 10% CuSO_4_ in 8% phosphoric acid and charring at 180 °C for 3 min [25]. For quantification, lipids on TLC plate were visualized with iodine vapor, and the silica gel corresponding to each lipid fraction was carefully scrapped off the TLC plate. Lipids were transesterified to fatty acid methyl esters (FAMEs) and analyzed by a gas chromatography–mass spectrometry (GC–MS) equipped with a DB-WAX capillary column (30 m × 0.25 mm × 0.25 μm) (Agilent, CA, USA). Helium was used as the carrier gas with the flow rate of 1.2 mL/min. The ion temperature and interface temperature were set at 200 °C and 240 °C, respectively. Samples were injected in split mode (19:1 split ratio) at an oven temperature of 45 °C with an injection volume of 1 μL. The oven temperature was raised to 150 °C at 15 °C min^−1^, then to 240 °C at 6 °C min^−1^ and held at 240 °C for 6 min. Total lipids were quantified as the fatty acids contained in the total lipids, namely total fatty acids (TFA), and TAG was quantified as the fatty acids in TAG.

### Statistical analysis

All experiments were determined in biological triplicate to ensure the reproducibility. Experimental results were obtained as the mean value ± SD. Statistical analyses were performed using the SPSS statistical package (SPSS Inc., Chicago, IL, USA). Paired-samples *t*-test was used for two group means. The statistical significances are achieved when *P* < 0.01.

## Additional files

**Additional file 1: Figure S1.** Validation of incorrect assembly of part of Chr09. **a**, **b** Part of Chr03 (**a**) and Chr09 (**b**) viewed in JBrowse. **c** Sequence blast and PCR validation. Chr03 and Chr09 share a 35-kb sequence, which encodes ten genes including *CzDGTT3* (*Cz03g14070+Cz03g14080 or Cz09g23010+Cz09g23020*). We inferred a wrong assembly of this part may occur for Chr03 or Chr09. Primer pairs were designed for Chr03 (f1+r1) and Chr09 (f2+r1), respectively; only the former gave the expected PCR product, while the latter had no product (**c**), indicating the incorrect assembly of Chr09. **Figure S2.** Comparison between the gene models of DGATs predicted from Roth et al. [31] and ours confirmed by 5’-RACE and sequencing. The gene models from Roth et al. [31] and us are on the top and bottom of each panel, respectively. The red arrows indicate the primers used for cloning the full-length coding sequence. Note that based on the sequence of Cz11921100, PCR using cDNA as the template gave no product. **Figure S3.** Phylogenetic analysis showing relationships among CzDGATs and various annotated DGATs from plants, animal, fungi and microalgae. Alignment of amino acid sequences was conducted using ClustalX 2.1. The tree was generated in the MEGA6.0 software using the maximum-likelihood method. Protein sequences were used for calculations. The percentages of bootstrap support that was calculated from 1000 bootstrap resamplings are shown on the branches. Bootstrap values >40% are shown above the branch. The sources and Genbank Accession numbers of DGATs are shown in the brackets. **Figure S4.** Predicated transmembrane domains for NoDGAT1A NoDGAT1B by TMHMM (V2.0, http://www.cbs.dtu.dk/services/TMHMM/). **Figure S5.** Yeast one-hybrid assay identified TFs bound to the promoter region of Cz*DGAT1A* and Cz*DGTT5*. (-) and (+) designate the negative and positive control, respectively. Blue indicates the binding between the promoter and TF. **Figure S6.** Genomic map of *CzDGAT1A* viewed in JBrowse (**a**) and predicted binding sites of MYB (**b**) and bZIP (**c**) at the 2.0-kb *CzDGAT1A* promoter. The underlined sequences in (**b**) and (**c**) indicate the 5’ of neighboring gene, Cz06g05020. The TFmatrixIDs were shown in Additional file 3: Data S1. **Figure S7.** Genomic map of *CzDGTT5* viewed in JBrowse (**a**) and predicted binding sites of MYB (**b**) and bZIP (**c**) at the 2.0-kb *CzDGTT5* promoter. The underlined sequences in (**b**) and (**c**) indicate the 3’ of neighboring gene, Cz09g27300. The TFmatrixIDs were shown in Additional file 3: Data S1. **Figure S8.** Gene expression of MYB1, bZIP3, *CzDGAT1A*, *CzDGTT5*, Cz06g05020 and Cz09g27300 at 0 and 12 h of ND. Cz06g05020 and Cz09g27300 are the neighboring genes of *CzDGAT1A* and *CzDGTT5*, respectively (Additional file 1: Figure S6 and Figure S7).

**Additional file 2: Table S1.** Genbank accession number and GSPs for 5’ -RACE of putative *CzDGAT*s. **Table S2.** Conserved domain analysis and subcellular localization prediction of *C. zofingiensis* DGATs. **Table S3.** Primers using for the cloning of full-length coding sequence of *C. zofingiensis DGAT*s.

**Additional file 3: Data S1.** Prediction of possible binding sites of MYB and bZIP at the promoter regions (2.0 kb) of *CzDGAT1A* and *CzDGTT5* using the software PlantPAN 2.0 (http://plantpan2.itps.ncku.edu.tw/).

## Abbreviations

CoA: coenzyme A
Cp: chloroplast
*C. zofingiensis*: *Chlorella zofingiensis*
DAG: diacylglycerol
DGAT: diacylglycerol acyltransferase
DGAT1: type I DGAT
DGAT2/DGTT: type II DGAT
DGAT3: type III DGAT
DGDG: digalactosyl diacylglycerol
ER: endoplasmic reticulum
EV: empty vector
FFA: free fatty acid
GC–MS: gas chromatography–mass spectrometry
GFP: green fluorescent protein
PCR: polymerase chain reaction
RACE: rapid amplification of cDNA ends
TAG: triacylglycerol
TF: transcription factor
TFA: total fatty acids
TLC: thin-layer chromatography
UTR: untranslated region
