# Supplementary material for: Characterization of type I and type II diacylglycerol acyltransferases from the emerging model alga Chlorella zofingiensis reveals their functional complementarity and engineering potential
Source: Biotechnol Biofuels. 2019 Feb 11;12:28. doi: 10.1186/s13068-019-1366-2 (PMC6371474; doi:10.1186/s13068-019-1366-2)
Supplement: Supplementary file 1 — Additional file 1: Figure S1. Validation of incorrect assembly of part of Chr09. a, b Part of Chr03 (a) and Chr09 (b) viewed in JBrowse. c Sequence blast and PCR validation. Chr03 and Chr09 share a 35-kb sequence, which encodes ten genes including CzDGTT3 (Cz03g14070+Cz03g14080 or Cz09g23010+Cz09g23020). We inferred a wrong assembly of this part may occur for Chr03 or Chr09. Primer pairs were designed for Chr03 (f1+r1) and Chr09 (f2+r1), respectively; only the former gave the expected PCR product, while the latter had no product (c), indicating the incorrect assembly of Chr09. Figure S2. Comparison between the gene models of DGATs predicted from Roth et al. [31] and ours confirmed by 5’-RACE and sequencing. The gene models from Roth et al. [31] and us are on the top and bottom of each panel, respectively. The red arrows indicate the primers used for cloning the full-length coding sequence. Note that based on the sequence of Cz11921100, PCR using cDNA as the template gave no product. Figure S3. Phylogenetic analysis showing relationships among CzDGATs and various annotated DGATs from plants, animal, fungi and microalgae. Alignment of amino acid sequences was conducted using ClustalX 2.1. The tree was generated in the MEGA6.0 software using the maximum-likelihood method. Protein sequences were used for calculations. The percentages of bootstrap support that was calculated from 1000 bootstrap resamplings are shown on the branches. Bootstrap values >40% are shown above the branch. The sources and Genbank Accession numbers of DGATs are shown in the brackets. Figure S4. Predicated transmembrane domains for NoDGAT1A NoDGAT1B by TMHMM (V2.0, http://www.cbs.dtu.dk/services/TMHMM/). Figure S5. Yeast one-hybrid assay identified TFs bound to the promoter region of CzDGAT1A and CzDGTT5. (-) and (+) designate the negative and positive control, respectively. Blue indicates the binding between the promoter and TF. Figure S6. Genomic map of CzDGAT1A viewed in JBrowse (a) and predict [file 13068_2019_1366_MOESM1_ESM.pdf]

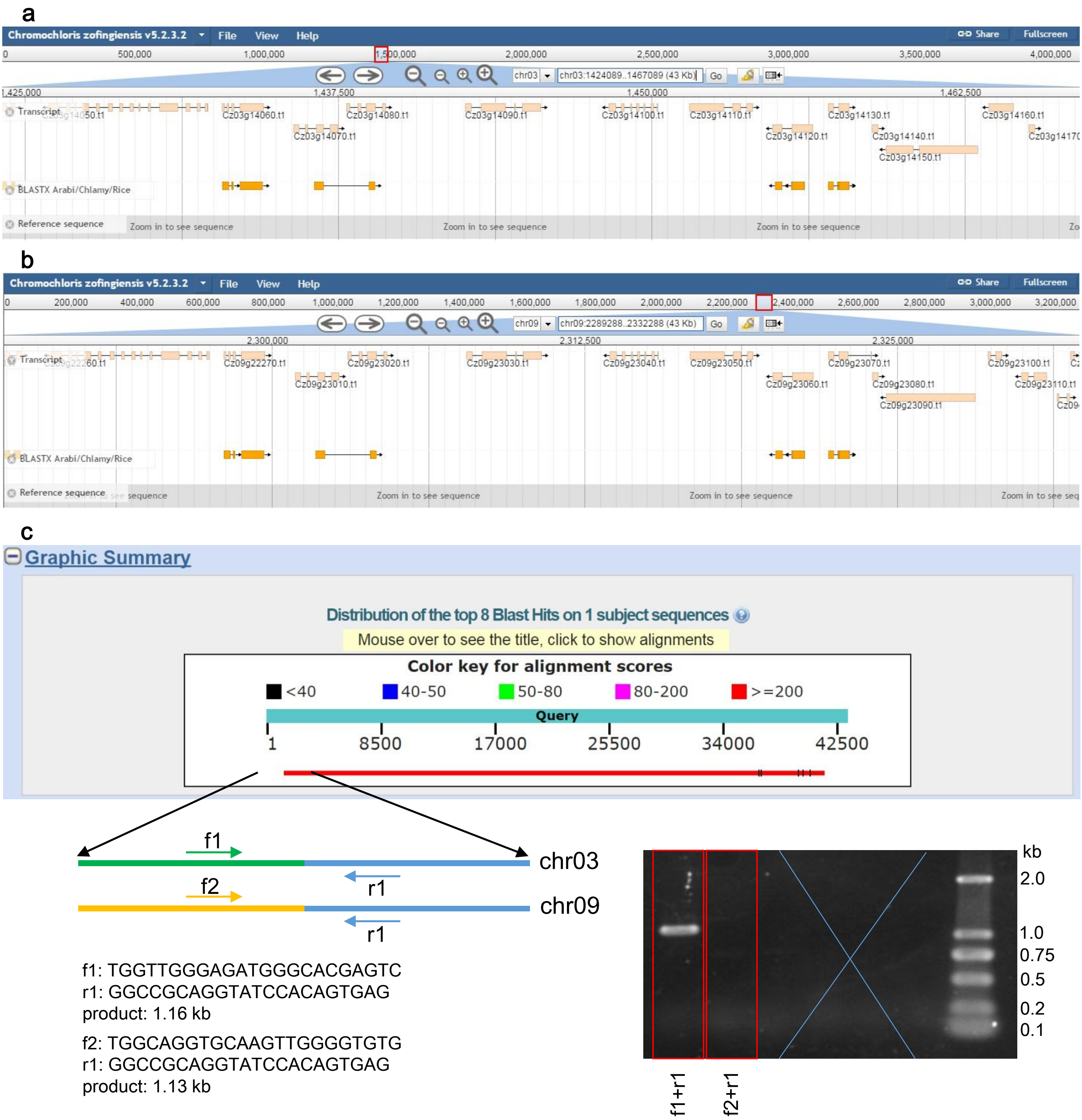

Figure S1. Validation of incorrect assembly of part of Chr09. **a**, **b** Part of Chr03 (**a**) and Chr09 (**b**) viewed in JBrowse. **c** Sequence blast and PCR validation. Chr03 and Chr09 share a 35-kb sequence, which encodes ten genes including *CzDGTT3* (*Cz03g14070+Cz03g14080 or Cz09g23010+Cz09g23020*). We inferred a wrong assembly of this part may occur for Chr03 or Chr09. Primer pairs were designed for Chr03 (f1+r1) and Chr09 (f2+r1), respectively; only the former gave the expected PCR product, while the latter had no product (**c**), indicating the incorrect assembly of Chr09.

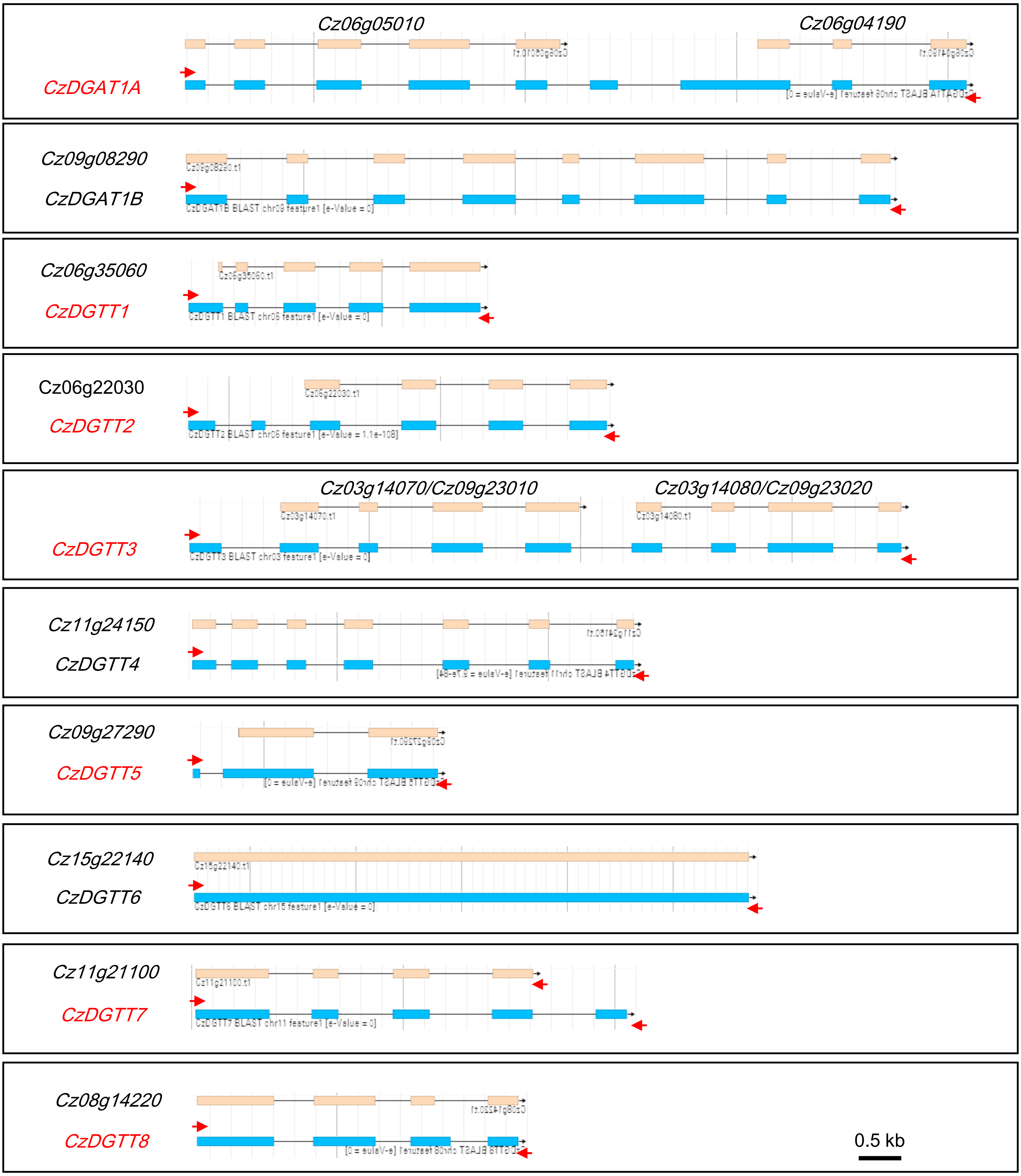

Figure S2. Comparison between the gene models of *DGAT*s predicted from Roth et al (2017) and ours confirmed by 5'-RACE and sequencing. The gene models from Roth et al. (2017) and us are on the top and bottom of each panel, respectively. The red arrows indicate the primers used for cloning the full-length coding sequence. Note that based on the sequence of Cz11921100, PCR using cDNA as the template gave no product.

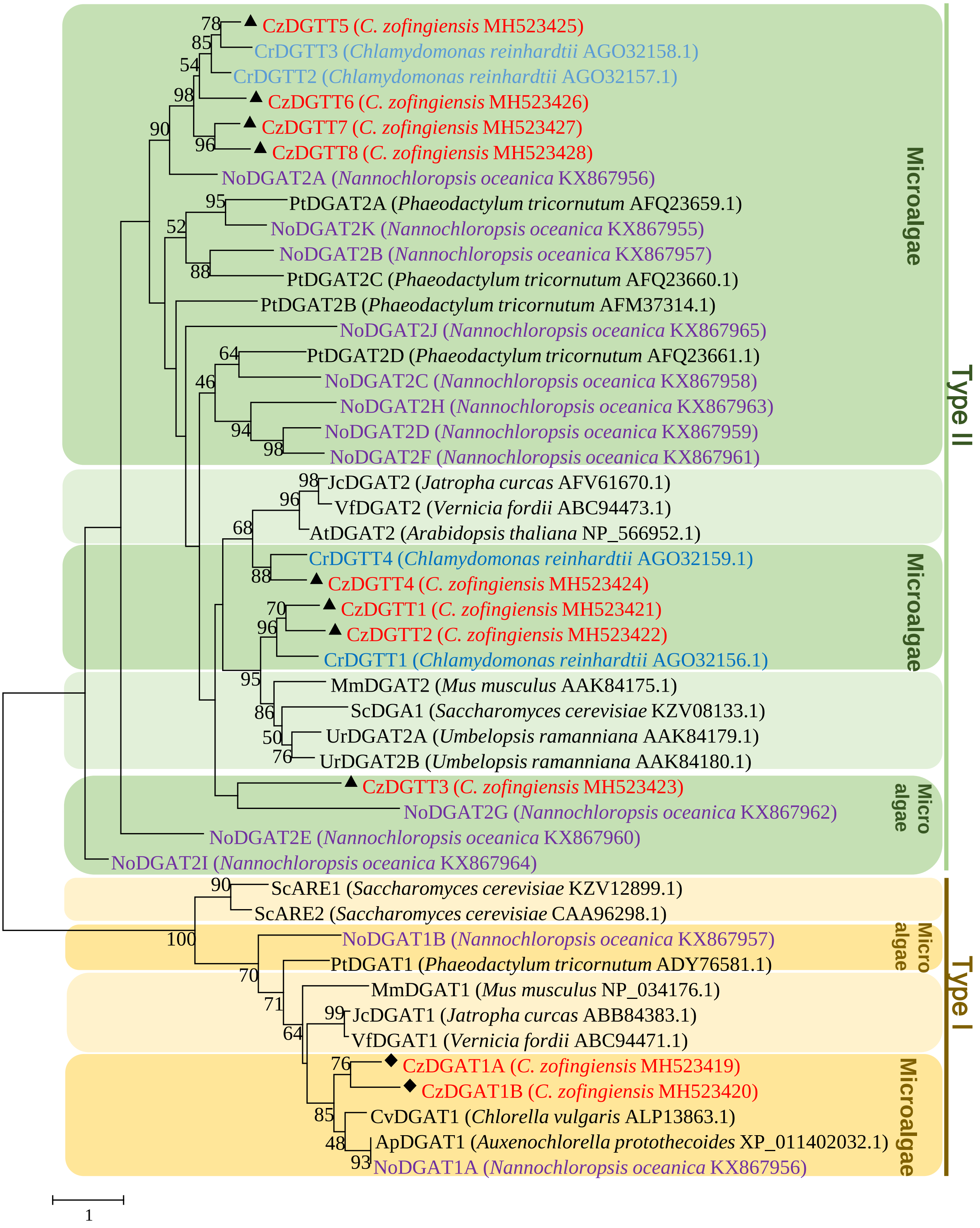

Figure S3. Phylogenetic analysis showing relationships among CzDGATs and various annotated DGATs from plants, animal, fungi and microalgae. Alignment of amino acid sequences was conducted using ClustalX 2.1. The tree was generated in the MEGA6.0 software using the maximum-likelihood method. Protein sequences were used for calculations. The percentages of bootstrap support that was calculated from 1000 bootstrap resamplings are shown on the branches. Bootstrap values >40% are shown above the branch. The sources and Genbank Accession numbers of DGATs are shown in the brackets.

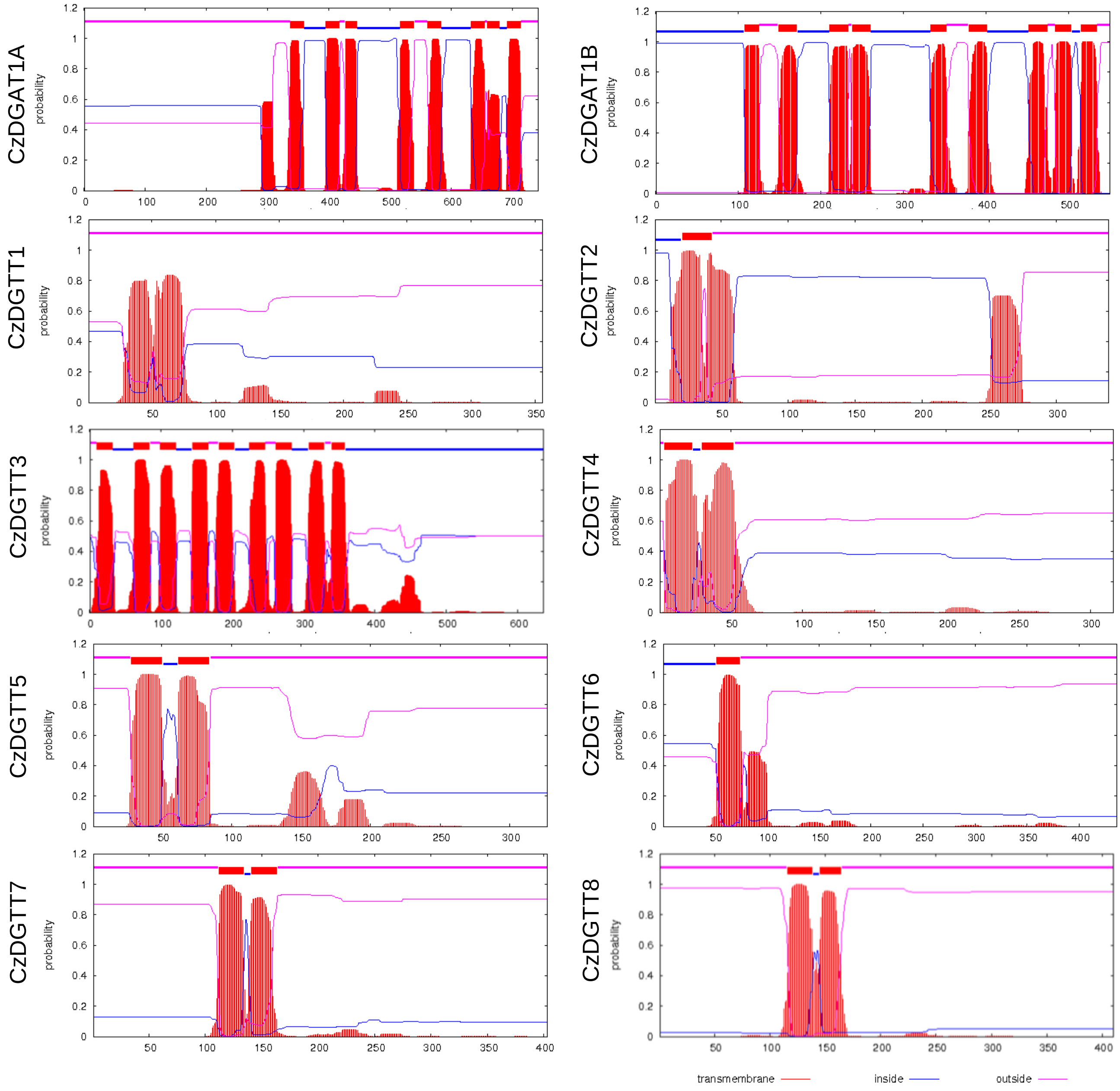

Figure S4. Predicated transmembrane domains for NoDGAT1A NoDGAT1B by TMHMM (V2.0, http://www.cbs.dtu.dk/services/TMHMM/).

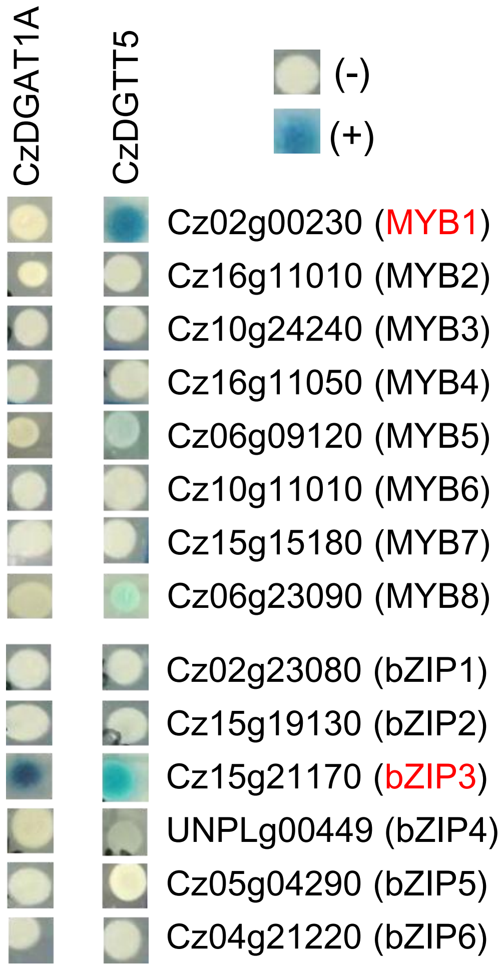

Figure S5. Yeast one-hybrid assay identified TFs bound to the promoter region of *czDGAT1A* and *CzDGTT5*. (-) and (+) designate the negative and positive control, respectively. Blue indicates the binding between the promoter and TF.

a

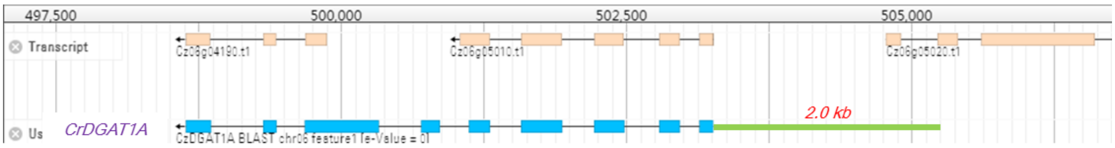

b

Predicted binding sites of MYB at the CzDGAT1A promoter

Strand + (5′-3′)

```
GTCACGTGCCCTACTAACTGCATCCAAGGCTTGCCTGCATCATGCGTTGAGCAACATCGCACATCAGCATGTCTGACATGGCTGTACACAATGAAAGAACCAGAATAAACACGAAGCAAGTTTTG
CATTAACATCAGAGTACATGCTGGTAGCACATCAGAGTATCAGGAAACAACGAAAGGTGTGGTGATGCATGGGCTGGCAGCCAGAAAAGGAAGTCAAATGCGGGAACAGCAAGCATGTGCTCTGC
TGCCTGCTAATTGCTAGACGGCATGCCCCTGCATATCAGTTGATGACGTAATGTTCTCAGACAACAATGCTCAGGGAGAAGCTGATGTGTGTGCTGTGTTACCTGCGCTCTTCCGCTAGCCGCCT
TGTACTGTACAGCTCAGTGCGCAACTTGCCAATTTCTGCTTGGGCTTCCTGCAGCATGAGCATATGCATGTGCTGCTCTGCGTGCTGCTCATCCACGCTGCCACTGTCCATGTTTGTGTGTCACA
CACCAGGGTGGGAACCTGCATGCACTAGACATGTGTGAGTGTTGCCTGGGAAAGCTGGATTGTACAGGAAGGAATGCTACATGGCTCCACAATGTATGGTGTATGTTGGTGAACTAGGAATCGTT
GTAAATCGTGATTCCAAAGGAGGGACAAAGCTAAGGGCCTCTGCTTGCTACACCATACAGGTCAGCAGTTGGTGCAGGCATACTTGTTGCAGTCACATGCGTGCTTGTGCAAATACCAGGTTAAC
TGCTAGGTACCAACAACATGCTGCCTGGCAGATTGATGCCTAACCTTATCAACTAGCAGGCCACAATCACTTGTAACCAGCACACCAGAGAACGTAGTTGACAAGTAAGATCACAGGCAACTGCT
AGTGTGCACAAGCTGAGACGCGGGGAGTAGAGGTGAGCACAACATCTGCCGTTGTTCGCCCATGATCGTGAGTTGTGGCCAGTGGGCGGGTTGGTGATGGGCATGCCACATCCAGTTCAGGTGCT
TGGTTCCCTCAACAAGACAGTGTTAACTATCAGCCATGCCATTGCACATGACCCGTAATAAGCAATGAGTTCCAAGCTATCACACGTGACTGTATGCTAAAAGACCAAAAGAGTCGAACATGTTC
AAAAGACGGTTGTTACAACAGTTGTCTCCGATAAGTCTTAGACCCTCGCTGCGTGGTAGCTCGTTGCCGTGACAGTACGCACTCACTATGAGTGCGGTCAGCAGCTTGAATAGCAGACATATGGG
CCAACATACACAGTACCACTATCAAGCCAGCCAGTACATATATGCTGGTATGCTGTGTGCACGGTTTACTTGTCTACAGTGGCGTGTTGCGACAGATCTATATAGTTGCAGAAACAGTTCGGTGC
CGTTGGTATCAAGTCAGGCACAAAGACTTTAACGCTCGCAATCGTGTACTGCATTTGACCAGTGTATACCAGTGTATAAGTAGTGTATCCTTTATGATGATCTGCTTGTCGGCAGCTTGCCATTG
TTTGAAAGGGCTCTGACAACCGGAACTTTAGCTGAGCACCACTGAAGCACATTGCCAGGTACCCTACTTTACAAGCTGACCGCTTCCACAATGCCGTATGGAGTTCTGCTCGCCATGCAATATAA
TATTCTGCCGCATGGGCAAATGCCCCCTAGTTTTTTGCGTTTTCACCAGACTGTACTATCTCCTTTGGGGTCCCTAAGTGCATGCACGCGTGCCTGTATACAGTCAAGTCAGTTACGTTGCGGAG
TTGTCATGACAATGTTCAGGGGATGTAGATATGCACTGCATTTGGCATGTTTGCCCAGATGGTGACACGAAAGTTCTTCGCTGGGGAATTCAGCTGCTAATAACTGCACGACTGTTGCATGGGCC
GCGGGCCGGTGTTGCATTGTTTTGACCTCGTCCTTCCGCTTCCTTTTGCTTATTGGTTTGTGTGACAATGCAGGCTAGCGATACCAATATATGCTTTACTGACTAAATGTCTGTTTACTGGCAGG
```

Strand – (3′-5′)

```
CAGTGCACGGGATGATTGACGTAGGTTCCGAACGGACGTAGTACGCAACTCGTTGTAGCGTGTAGTCGTACAGACTGTACCGACATGTGTTACTTTCTTGGTCTTATTTGTGCTTCGTTCAAAAC
GTAATTGTAGTCTCATGTACGACCATCGTGTAGTCTCATAGTCCTTTGTTGCTTTCCACACCACTACGTACCCGACCGTCGGTCTTTTCCTTCAGTTTACGCCCTTGTCGTTCGTACACGAGACG
ACGGACGATTAACGATCTGCCGTACGGGGACGTATAGTCAACTACTGCATTACAAGAGTCTGTTGTTACGAGTCCCTCTTCGACTACACACACGACACAATGGACGCGAGAAGGCGATCGGCGGA
ACATGACATGTCGAGTCACGCGTTGAACGGTTAAAGACGAACCCGAAGGACGTCGTACTCGTATACGTACACGACGAGACGCACGACGAGTAGGTGCGACGGTGACAGGTACAAACACACAGTGT
GTGGTCCCACCCTTGGACGTACGTGATCTGTACACACTCACAACGGACCCTTTCGACCTAACATGTCCTTCCTTACGATGTACCGAGGTGTTACATACCACATACAACCACTTGATCCTTAGCAA
CATTTAGCACTAAGGTTTCCTCCCTGTTTCGATTCCCGGAGACGAACGATGTGGTATGTCCAGTCGTCAACCACGTCCGTATGAACAACGTCAGTGTACGCACGAACACGTTTATGGTCCAATTG
ACGATCCATGGTTGTTGTACGACGGACCGTCTAACTACGGATTGGAATAGTTGATCGTCCGGTGTTAGTGAACATTGGTCGTGTGGTCTCTTGCATCAACTGTTCATTCTAGTGTCCGTTGACGA
TCACACGTGTTCGACTCTGCGCCCCTCATCTCCACTCGTGTTGTAGACGGCAACAAGCGGGTACTAGCACTCAACACCGGTCACCCGCCCAACCACTACCCGTACGGTGTAGGTCAAGTCCACGA
ACCAAGGGAGTTGTTCTGTCACAATTGATAGTCGGTACGGTAACGTGTACTGGGCATTATTCGTTACTCAAGGTTCGATAGTGTGCACTGACATACGATTTTCTGGTTTTCTCAGCTTGTACAAG
TTTTCTGCCAACAATGTTGTCAACAGAGGCTATTCAGAATCTGGGAGCGACGCACCATCGAGCAACGGCACTGTCATGCGTGAGTGATACTCACGCCAGTCGTCGAACTTATCGTCTGTATACCC
GGTTGTATGTGTCATGGTGATAGTTCGGTCGGTCATGTATATACGACCATACGACACACGTGCCAAATGAACAGATGTCACCGCACAACGCTGTCTAGATATATCAACGTCTTTGTCAAGCCACG
GCAACCATAGTTCAGTCCGTGTTTCTGAAATTGCGAGCGTTAGCACATGACGTAAACTGGTCACATATGGTCACATATTCATCACATAGGAAATACTACTAGACGAACAGCCGTCGAACGGTAAC
AAACTTTCCCGAGACTGTTGGCCTTGAAATCGACTCGTGGTGACTTCGTGTAACGGTCCATGGGATGAAATGTTCGACTGGCGAAGGTGTTACGGCATACCTCAAGACGAGCGGTACGTTATATT
ATAAGACGGCGTACCCGTTTACGGGGGATCAAAAAACGCAAAAGTGGTCTGACATGATAGAGGAAACCCCAGGGATTCACGTACGTGCGCACGGACATATGTCAGTTCAGTCAATGCAACGCCTC
AACAGTACTGTTACAAGTCCCCTACATCTATACGTGACGTAAACCGTACAAACGGGTCTACCACTGTGCTTTCAAGAAGCGACCCCTTAAGTCGACGATTATTGACGTGCTGACAACGTACCCGG
CGCCCGGCCACAACGTAACAAAACTGGAGCAGGAAGGCGAAGGAAAACGAATAACCAAACACACTGTTACGTCCGATCGCTATGGTTATATACGAAATGACTGATTTACAGACAAATGACCGTCC
```

TFmatrixID_0030: TFmatrixID_0048: TFmatrixID_0333: TFmatrixID_0342: TFmatrixID_0345: TFmatrixID_0347: TFmatrixID_0353:
TFmatrixID_0357: TFmatrixID_0362: TFmatrixID_0363: TFmatrixID_0486: TFmatrixID_0610: TF_motif_seq_0252:

c

Predicted binding sites of bZIP at the CzDGAT1A promoter

Strand + (5′-3′)

```
GTCACGTGCCCTACTAACTGCATCCAAGGCTTGCCTGCATCATGCGTTGAGCAACATCGCACATCAGCATGTCTGACATGGCTGTACACAATGAAAGAACCAGAATAAACACGAAGCAAGTTTTG
CATTAACATCAGAGTACATGCTGGTAGCACATCAGAGTATCAGGAAACAACGAAAGGTGTGGTGATGCATGGGCTGGCAGCCAGAAAAGGAAGTCAAATGCGGGAACAGCAAGCATGTGCTCTGC
TGCCTGCTAATTGCTAGACGGCATGCCCCTGCATATCAGTTGATGACGTAATGTTCTCAGACAACAATGCTCAGGGAGAAGCTGATGTGTGTGCTGTGTTACCTGCGCTCTTCCGCTAGCCGCCT
TGTACTGTACAGCTCAGTGCGCAACTTGCCAATTTCTGCTTGGGCTTCCTGCAGCATGAGCATATGCATGTGCTGCTCTGCGTGCTGCTCATCCACGCTGCCACTGTCCATGTTTGTGTGTCACA
CACCAGGGTGGGAACCTGCATGCACTAGACATGTGTGAGTGTTGCCTGGGAAAGCTGGATTGTACAGGAAGGAATGCTACATGGCTCCACAATGTATGGTGTATGTTGGTGAACTAGGAATCGTT
GTAAATCGTGATTCCAAAGGAGGGACAAAGCTAAGGGCCTCTGCTTGCTACACCATACAGGTCAGCAGTTGGTGCAGGCATACTTGTTGCAGTCACATGCGTGCTTGTGCAAATACCAGGTTAAC
TGCTAGGTACCAACAACATGCTGCCTGGCAGATTGATGCCTAACCTTATCAACTAGCAGGCCACAATCACTTGTAACCAGCACACCAGAGAACGTAGTTGACAAGTAAGATCACAGGCAACTGCT
AGTGTGCACAAGCTGAGACGCGGGGAGTAGAGGTGAGCACAACATCTGCCGTTGTTCGCCCATGATCGTGAGTTGTGGCCAGTGGGCGGGTTGGTGATGGGCATGCCACATCCAGTTCAGGTGCT
TGGTTCCCTCAACAAGACAGTGTTAACTATCAGCCATGCCATTGCACATGACCCGTAATAAGCAATGAGTTCCAAGCTATCACACGTGACTGTATGCTAAAAGACCAAAAGAGTCGAACATGTTC
AAAAGACGGTTGTTACAACAGTTGTCTCCGATAAGTCTTAGACCCTCGCTGCGTGGTAGCTCGTTGCCGTGACAGTACGCACTCACTATGAGTGCGGTCAGCAGCTTGAATAGCAGACATATGGG
CCAACATACACAGTACCACTATCAAGCCAGCCAGTACATATATGCTGGTATGCTGTGTGCACGGTTTACTTGTCTACAGTGGCGTGTTGCGACAGATCTATATAGTTGCAGAAACAGTTCGGTGC
CGTTGGTATCAAGTCAGGCACAAAGACTTTAACGCTCGCAATCGTGTACTGCATTTGACCAGTGTATACCAGTGTATAAGTAGTGTATCCTTTATGATGATCTGCTTGTCGGCAGCTTGCCATTG
TTTGAAAGGGCTCTGACAACCGGAACTTTAGCTGAGCACCACTGAAGCACATTGCCAGGTACCCTACTTTACAAGCTGACCGCTTCCACAATGCCGTATGGAGTTCTGCTCGCCATGCAATATAA
TATTCTGCCGCATGGGCAAATGCCCCCTAGTTTTTTGCGTTTTCACCAGACTGTACTATCTCCTTTGGGGTCCCTAAGTGCATGCACGCGTGCCTGTATACAGTCAAGTCAGTTACGTTGCGGAG
TTGTCATGACAATGTTCAGGGGATGTAGATATGCACTGCATTTGGCATGTTTGCCCAGATGGTGACACGAAAGTTCTTCGCTGGGGAATTCAGCTGCTAATAACTGCACGACTGTTGCATGGGCC
GCGGGCCGGTGTTGCATTGTTTTGACCTCGTCCTTCCGCTTCCTTTTGCTTATTGGTTTGTGTGACAATGCAGGCTAGCGATACCAATATATGCTTTACTGACTAAATGTCTGTTTACTGGCAGG
```

Strand – (3′-5′)

```
CAGTGCACGGGATGATTGACGTAGGTTCCGAACGGACGTAGTACGCAACTCGTTGTAGCGTGTAGTCGTACAGACTGTACCGACATGTGTTACTTTCTTGGTCTTATTTGTGCTTCGTTCAAAAC
GTAATTGTAGTCTCATGTACGACCATCGTGTAGTCTCATAGTCCTTTGTTGCTTTCCACACCACTACGTACCCGACCGTCGGTCTTTTCCTTCAGTTTACGCCCTTGTCGTTCGTACACGAGACG
ACGGACGATTAACGATCTGCCGTACGGGGACGTATAGTCAACTACTGCATTACAAGAGTCTGTTGTTACGAGTCCCTCTTCGACTACACACACGACACAATGGACGCGAGAAGGCGATCGGCGGA
ACATGACATGTCGAGTCACGCGTTGAACGGTTAAAGACGAACCCGAAGGACGTCGTACTCGTATACGTACACGACGAGACGCACGACGAGTAGGTGCGACGGTGACAGGTACAAACACACAGTGT
GTGGTCCCACCCTTGGACGTACGTGATCTGTACACACTCACAACGGACCCTTTCGACCTAACATGTCCTTCCTTACGATGTACCGAGGTGTTACATACCACATACAACCACTTGATCCTTAGCAA
CATTTAGCACTAAGGTTTCCTCCCTGTTTCGATTCCCGGAGACGAACGATGTGGTATGTCCAGTCGTCAACCACGTCCGTATGAACAACGTCAGTGTACGCACGAACACGTTTATGGTCCAATTG
ACGATCCATGGTTGTTGTACGACGGACCGTCTAACTACGGATTGGAATAGTTGATCGTCCGGTGTTAGTGAACATTGGTCGTGTGGTCTCTTGCATCAACTGTTCATTCTAGTGTCCGTTGACGA
TCACACGTGTTCGACTCTGCGCCCCTCATCTCCACTCGTGTTGTAGACGGCAACAAGCGGGTACTAGCACTCAACACCGGTCACCCGCCCAACCACTACCCGTACGGTGTAGGTCAAGTCCACGA
ACCAAGGGAGTTGTTCTGTCACAATTGATAGTCGGTACGGTAACGTGTACTGGGCATTATTCGTTACTCAAGGTTCGATAGTGTGCACTGACATACGATTTTCTGGTTTTCTCAGCTTGTACAAG
TTTTCTGCCAACAATGTTGTCAACAGAGGCTATTCAGAATCTGGGAGCGACGCACCATCGAGCAACGGCACTGTCATGCGTGAGTGATACTCACGCCAGTCGTCGAACTTATCGTCTGTATACCC
GGTTGTATGTGTCATGGTGATAGTTCGGTCGGTCATGTATATACGACCATACGACACACGTGCCAAATGAACAGATGTCACCGCACAACGCTGTCTAGATATATCAACGTCTTTGTCAAGCCACG
GCAACCATAGTTCAGTCCGTGTTTCTGAAATTGCGAGCGTTAGCACATGACGTAAACTGGTCACATATGGTCACATATTCATCACATAGGAAATACTACTAGACGAACAGCCGTCGAACGGTAAC
AAACTTTCCCGAGACTGTTGGCCTTGAAATCGACTCGTGGTGACTTCGTGTAACGGTCCATGGGATGAAATGTTCGACTGGCGAAGGTGTTACGGCATACCTCAAGACGAGCGGTACGTTATATT
ATAAGACGGCGTACCCGTTTACGGGGGATCAAAAAACGCAAAAGTGGTCTGACATGATAGAGGAAACCCCAGGGATTCACGTACGTGCGCACGGACATATGTCAGTTCAGTCAATGCAACGCCTC
AACAGTACTGTTACAAGTCCCCTACATCTATACGTGACGTAAACCGTACAAACGGGTCTACCACTGTGCTTTCAAGAAGCGACCCCTTAAGTCGACGATTATTGACGTGCTGACAACGTACCCGG
CGCCCGGCCACAACGTAACAAAACTGGAGCAGGAAGGCGAAGGAAAACGAATAACCAAACACACTGTTACGTCCGATCGCTATGGTTATATACGAAATGACTGATTTACAGACAAATGACCGTCC
```

TFmatrixID_0182: TFmatrixID_0183: TFmatrixID_0184: TFmatrixID_0185: TFmatrixID_0187: TFmatrixID_0188: TFmatrixID_0189:
TFmatrixID_0191: TFmatrixID_0195: TFmatrixID_0196: TFmatrixID_0197: TFmatrixID_0199: TFmatrixID_0202: TFmatrixID_0203:
TFmatrixID_0490: TFmatrixID_0533: TFmatrixID_0620: TF_motif_seq_0240: TF_motif_seq_0271: TF_motif_seq_0287:

Figure S6. Genomic map of *CzDGAT1A* viewed in JBrowse (**a**) and predicted binding sites of MYB (**b**) and bZIP (**c**) at the 2.0-kb *CzDGAT1A* promoter. The underlined sequences in (**b**) and (**c**) indicate the 5’ of neighboring gene, Cz06g05020. The TFmatrixIDs were shown in Additional file 3: Data S1.

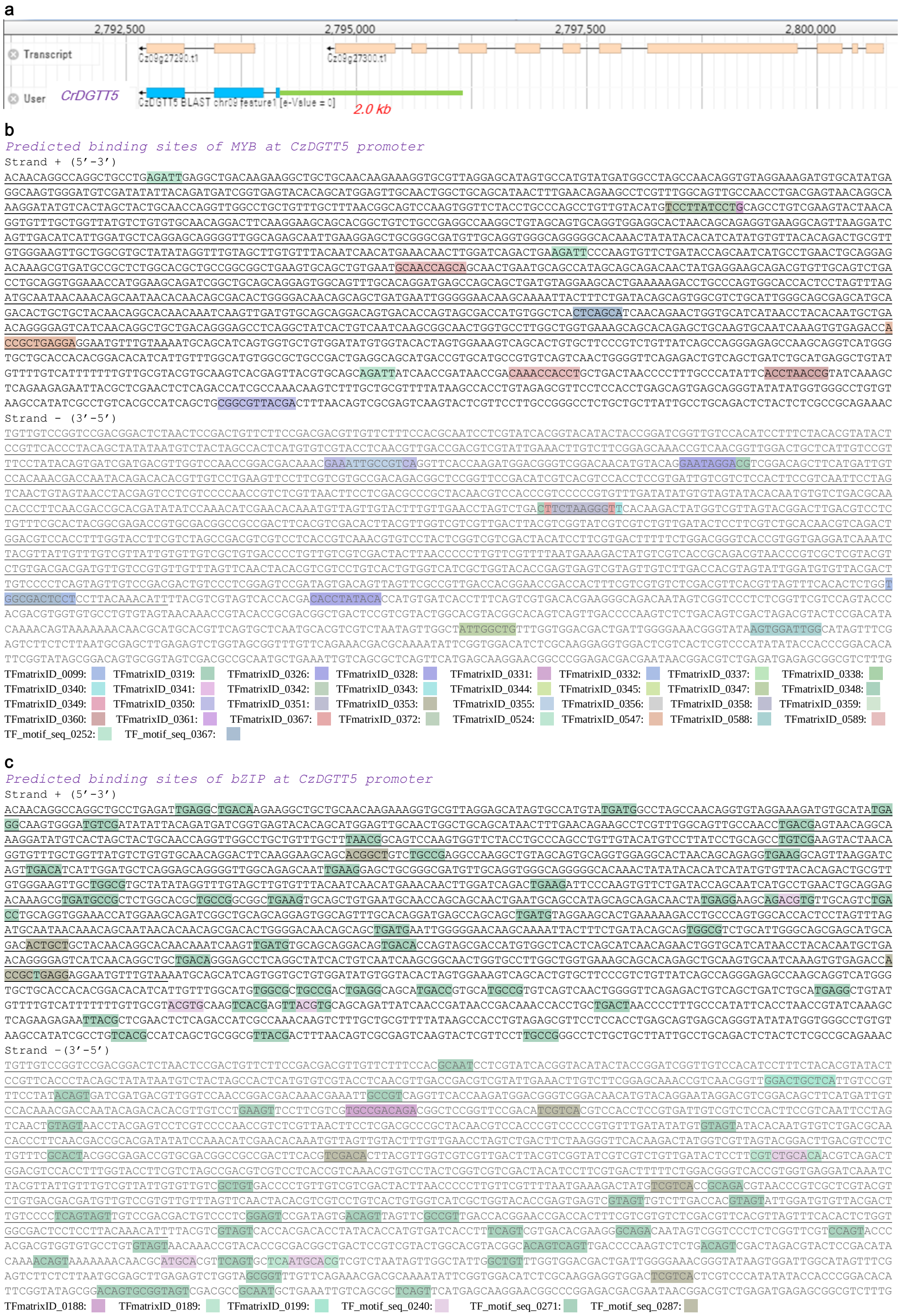

Figure S7. Genomic map of *CzDGTT5* viewed in JBrowse (**a**) and predicted binding sites of MYB (**b**) and bZIP (**c**) at the 2.0-kb *CzDGTT5* promoter. The underlined sequences in (**b**) and (**c**) indicate the 3' of neighboring gene, Cz09g27300. The TFmatrixIDs were shown in Additional file 3: Data S1.

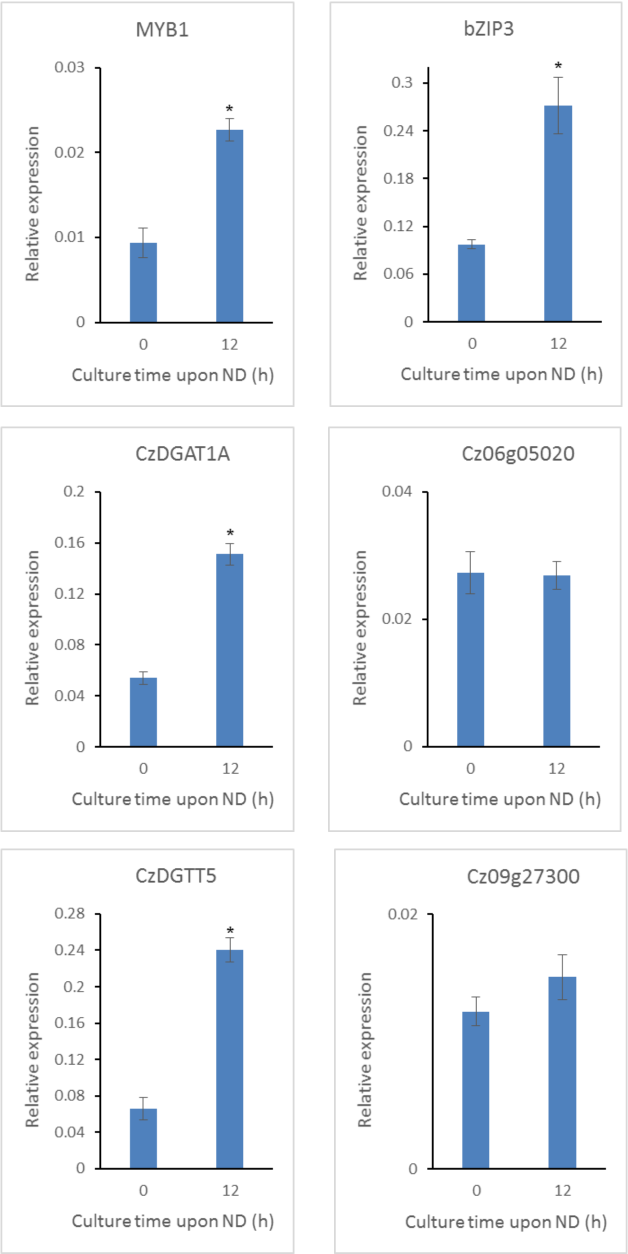

Figure S8. Gene expression of MYB1, bZIP3, *CzDGAT1A*, *CzDGTT5*, Cz06g05020 and Cz09g27300 at 0 and 12 h of ND. Cz06g05020 and Cz09g27300 are the 5' neighboring genes of *CzDGAT1A* and *CzDGTT5*, respectively (Additional file 1: Figure S6 and Figure S7). Asterisks indicate the significant difference compared to 0 h (*t*-test, $P < 0.01$).
