# Supplementary material for: Characterization of type I and type II diacylglycerol acyltransferases from the emerging model alga Chlorella zofingiensis reveals their functional complementarity and engineering potential
Source: Biotechnol Biofuels. 2019 Feb 11;12:28. doi: 10.1186/s13068-019-1366-2 (PMC6371474; doi:10.1186/s13068-019-1366-2)
Supplement: Supplementary file 2 — Additional file 2: Table S1. Genbank accession number and GSPs for 5’ -RACE of putative CzDGATs. Table S2. Conserved domain analysis and subcellular localization prediction of C. zofingiensis DGATs. Table S3. Primers using for the cloning of full-length coding sequence of C. zofingiensis DGATs. [file 13068_2019_1366_MOESM2_ESM.docx]

**Table S1**. Genbank accession number and gene specific primers for 5’ -RACE of putative *C. zofingiensis* *DGAT*s

| Gene Name | Genbank accession number | Gene IDs in Roth et al (2017) ^1^ | Gene specific primers (GSPs) | |
| --- | --- | --- | --- | --- |
|  |  |  | First round GSP (5’-3’) | Second round Nest-GSP (5’-3’) |
| CzDGAT1A | MH523419 | Cz06g04190 | CCACAATGCGAAACCGCCACTGCTG | TTCCACAAGACAATTCTGCACG |
| CzDGAT1B | MH523420 | Cz09g08290 | CAAAGATGACCACCGCCAAGTTGACCAG | GTCCATCATGCTTCTCTGTCCAC |
| CzDGTT1 | MH523421 | Cz06g35060 | TGAGCAGGCAGTACTCACGCACCAGTGG | TTGAAGTTGGATGATAGTGTCATGG |
| CzDGTT2 | MH523422 | Cz06g22030 | CCTTACCACTGCCTTGCCCCAACACCAT | CACACACCATGTAGCAACAGGAAC |
| CzDGTT3 | MH523423 | Cz09g23020  Cz03g14080 | ATTCCGGTCGCTTGCATCATACACCAGC | GCCCCTGATATGACATCCACAT |
| CzDGTT4 | MH523424 | Cz11g24150 | GCAGGGCTGCAGGCAATAAGGGTGACTC | AGGCAGAGACGAATGTGGCTC |
| CzDGTT5 | MH523425 | Cz09g27290 | ATGGCTTGACACTGAGTGCCAGCGATCAG | CTCACGCCATGTCTGGAACAC |
| CzDGTT6 | MH523426 | Cz15g22140 | AGAAGTGGAAATAACGTCGCCAGCAAAGG | ATAGCTGTCGAGAGCTTTTCGC |
| CzDGTT7 | MH523427 | Cz11g21100 | CCAGACCAGAAGGGCAGTCAGCACATGT | AATTGGTCTGCATACTGGCGTAC |
| CzDGTT8 | MH523428 | Cz08g14220 | GAACCGCCAATATGGATGTTCGGAGGCT | TAGCGCACACCAGTCTCGTACT |

^1^ Roth MS, Cokus SJ, Gallaher SD, Walter A, Lopez D, Erickson E, et al. Chromosome-level genome assembly and transcriptome of the green alga *Chromochloris zofingiensis* illuminates astaxanthin production. Proc Natl Aca Sci. 2017;114:4296-4305.

**Table S2.** Conserved domain analysis and subcellular localization prediction of *C. zofingiensis* DGATs

| Protein ID | Length | Conserved domain | Subcellular predication | | |
| --- | --- | --- | --- | --- | --- |
|  |  |  | PredAlgo | TargetP | ChloroP |
| CzDGAT1A | 743 | MBOAT superfamily, PH | O | O | N |
| CzDGAT1B | 550 | MBOAT superfamily | O | O | N |
| CzDGTT1 | 356 | DGAT, putative acyl-acceptor binding pocket | O | O | N |
| CzDGTT2 | 339 | DGAT, putative acyl-acceptor binding pocket | S | S | N |
| CzDGTT3 | 636 | DGAT, putative acyl-acceptor binding pocket | S | S | N |
| CzDGTT4 | 316 | DGAT, putative acyl-acceptor binding pocket | S | S | N |
| CzDGTT5 | 327 | DGAT, putative acyl-acceptor binding pocket | S | O | N |
| CzDGTT6 | 436 | DGAT, putative acyl-acceptor binding pocket | O | O | N |
| CzDGTT7 | 403 | DGAT, putative acyl-acceptor binding pocket | O | O | N |
| CzDGTT8 | 410 | DGAT, putative acyl-acceptor binding pocket | O | O | N |

MBOAT, membrane bound O-acyl transferase; PH, Pleckstrin homology domain;

PredAlgo and TargetP were used for multi-subcellular localization prediction: mitochondrion (M), chloroplast (C), and secretory pathway (S) or Other (O). ChloroP was used for predicting the presence of chloroplast transit peptides (cTP) in protein sequences: Y and N designate the presence and absence of predicted cTP, respectively.

**Table S3**. Primers using for the cloning of full-length coding sequence of *C. zofingiensis* *DGAT*s.

| Gene Name | Length (bp) | Forward and reverse primer (5’-3’) | Restriction enzyme |
| --- | --- | --- | --- |
| CzDGAT1A | 2232 | CCGGAATTCATGAAACTGGGCGCTCTG  CCGCTCGAGTCAGAAGCTAGGTATGGTGCTGTTG | EcoRI/ Xhol |
| CzDGAT1B | 1653 | CGAGCTCATGGAGGGTGCACGAATC  TGCTCTAGATCAGTGTGACATAAGAGCAGCATT | Sacl/XbaI |
| CzDGTT1 | 1071 | CCGGAATTCATGCAACGCATCTCTTGC  CCGCTCGAGAGTGTCTCACTCCACTATGGTAAG | EcoRI/ Xhol |
| CzDGTT2 | 1020 | CGAGCTCATGGCGAAACAAGCACTTGAT  TGCTCTAGACTACTCCACAATGCGCAAGCT | Sacl/XbaI |
| CzDGTT3 | 1911 | CCGGAATTCGGATGGACCTCATAGCGTTTC  CCGCTCGAGTTAGTCCTTTACCAGGATGAGATTAG | EcoRI/ Xhol |
| CzDGTT4 | 951 | CCGGAATTCATGAAGGACGCGGTAG  CCGCTCGAGCTATCTGATGAATAGCTGC | EcoRI/ Xhol |
| CzDGTT5 | 984 | CGAGCTCATGTTGGACACGGGTACGG  TGCTCTAGATCACTCAATCTCCAATGGCC | Sacl/XbaI |
| CzDGTT6 | 1311 | CCGGAATTCATGGGTCCAATCAGCAG  CCGCTCGAGTTACGTGATGACTAAAGGCCG | EcoRI/ Xhol |
| CzDGTT7 | 1212 | CGAGCTCATGGCCATGCCGGCAAAC  TGCTCTAGATCATGATGAGTGGTC | Sacl/XbaI |
| CzDGTT8 | 1233 | CGAGCTCATGGGACTTGGCCCAGG  TGCTCTAGATTACTAATGAATAATCAATGGCCTGT | Sacl/XbaI |
